# Cyclization of *ortho*-hydroxycinnamates to coumarins under mild conditions: A nucleophilic organocatalysis approach

**DOI:** 10.3762/bjoc.8.186

**Published:** 2012-09-26

**Authors:** Florian Boeck, Max Blazejak, Markus R Anneser, Lukas Hintermann

**Affiliations:** 1 Department Chemie, Technische Universität München, Lichtenbergstr. 4, 85748 Garching, Germany

**Keywords:** catalysis, coumarins, heterocycles, mechanisms, organocatalysis, phosphanes

## Abstract

(*E*)-Alkyl *ortho*-hydroxycinnamates cyclize to coumarins at elevated temperatures of 140–250 °C. We find that the use of tri-*n*-butylphosphane (20 mol %) as a nucleophilic organocatalyst in MeOH solution allows cyclization to take place under much milder conditions (60–70 °C). Several coumarins were prepared, starting from *ortho*-hydroxyarylaldehydes, by Wittig reaction with Ph_3_P=CHCO_2_Me to (*E*)-methyl *ortho*-hydroxycinnamates, followed by the phosphane catalyzed cyclization.

## Introduction

**Scheme 1:**
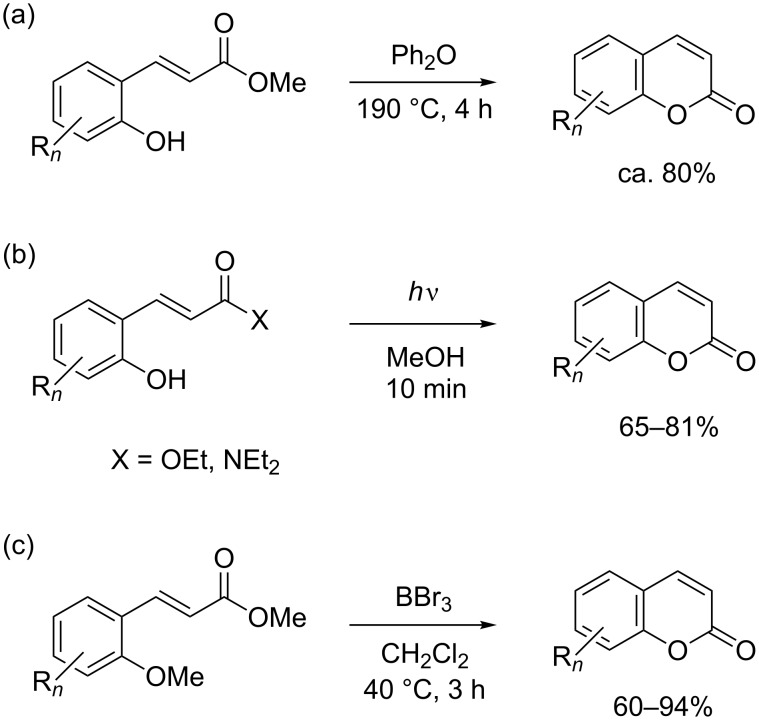
Conditions for the cyclization of 2’-hydroxycinnamate and related precursors to coumarins. (a) Thermal cyclization [16]. (b) Photochemical isomerization/cyclization [17]. (c) Lewis acid induced demethylation/cyclization [18].

Coumarins are important structural motifs in natural products and bioactive compounds, in which they exhibit broad biological activity, e.g., as anticoagulants, antifungal agents, antioxidants, or as anthelmintic, hypnotic and cytotoxic agents [1–4]. Due to their fluorescent properties, coumarins are also widely used as agrochemicals, additives in cosmetics and food, optical brighteners, and dispersed fluorescent and tunable laser-dye optical agents [5–6]. Classical synthetic approaches for coumarins are based on the Perkin reaction or von Pechmann condensation, i.e., reactions under harsh conditions and at elevated temperatures [1]. Recent new methodologies based on CH-activation reactions still use acidic reaction media and show, in part, a lack of regioselectivity [7–10]. Such problems may be circumvented by using ring-closing metathesis [11–12] or other approaches [13–15]. In selective synthetic schemes, the generation of coumarins is typically realized by the cyclization of *ortho*-hydroxycinnamates (Scheme 1).

This reaction requires high temperatures (140–250 °C, Scheme 1a) [16,19–29] or photochemical double-bond isomerization (Scheme 1b) [15,17,30–31]. An alternative boron tribromide induced lactonization proceeds at a lower temperature (Scheme 1c), but is not compatible with acid-sensitive functionality [18,32]. It follows that synthetic methods converting hydroxycinnamates to coumarins in the absence of acid under mild conditions are very desirable, particularly for labile starting materials, as often found in the late stages of multistep natural-product syntheses.

**Scheme 2:**
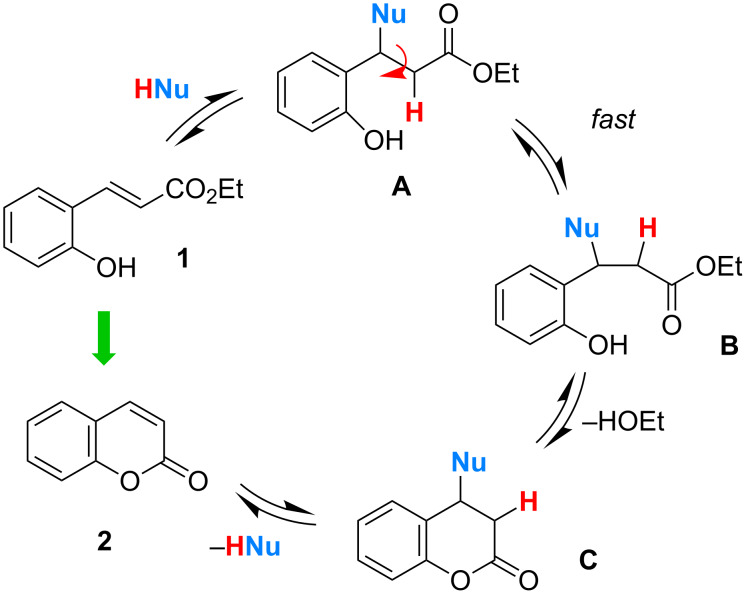
Hypothetical catalytic cycle: Nucleophile-assisted cyclization of (*E*)-ethyl 2’-hydroxycinnamate (**1**) to coumarin (**2**).

The difficulty of cyclizing (*E*)-2’-hydroxycinnamates to coumarins can be traced to the (*E*)-configuration of the starting material, which places the ester carbonyl group out of reach of the phenolic nucleophile [33–35]. The starting material must first be isomerized to a (*Z*)-configured intermediate, before cyclization can occur in a geometrically favored manner, but there is a considerable kinetic and energetic barrier against this isomerization process, provoking the observed high reaction temperatures. We wondered whether this problem could be circumvented by adding a nucleophile (HNu) to the reaction mixture containing **1**, which is capable of undergoing a reversible conjugate addition to form an intermediate **A** devoid of an alkene functionality (Scheme 2).

Rotation around the single bond to give **B** should be a fast process, and cyclization to a 2-chromanone **C** is then entropically favored. Eventually, the elimination to coumarin (**2**) could be driven by aromatic stabilization (Scheme 2). In fact, a related stoichiometric two-step protocol has been proposed [36]. It appeared to us that the practical problem of developing a mild and convenient catalytic conversion of *ortho*-hydroxycinnamates to the corresponding coumarins could be an ideal test case to show the utility of using organocatalytic rationales for solving a synthetic problem.

## Results and Discussion

The reaction of (*E*)-ethyl 2’-hydroxycinnamate (**1**) to coumarin (**2**) was chosen as the assay to find catalytic activity under mild conditions (Table 1).

**Table 1:** Screening of catalysts for nucleophilic double-bond isomerization.^a^

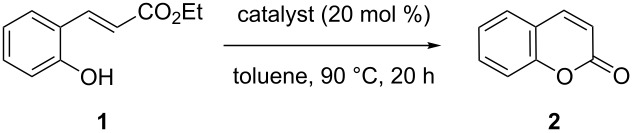		

Entry	Catalyst	Yield (%)^b^

1	PhSH	–
2	DABCO	–
3	cinchonine	–
4	DBU	–
5	DMAP	–
6	IMes^c^	<1
7	PPh_3_	–
8	*n*-Bu_3_P	89

^a^Reactions were performed on 0.5 mmol scale in 1 mL of solvent; reaction time 20–23 h. ^b^Screening yields were determined by GC and/or HPLC analysis with *N*-pivaloylaniline as internal standard. ^c^IMes was generated in situ from IMes·HCl [37] and DBU.

It was initially thought that hydro-heteroatomic nucleophiles such as thiols, which are known to easily undergo hetero-Michael additions [38], could be suitable candidates for the screen. However, coumarin was not formed in the presence of thiols (Table 1, entry 1). We turned our attention to nucleophiles that are established catalysts in Morita–Baylis–Hillman type reactions, where they add to conjugated acceptor systems [39]. Tests with either nitrogen bases (Table 1, entries 2–5) or N-heterocyclic carbenes (Table 1, entry 6) were not rewarded with success. Finally, phosphanes were tested, and while triphenylphosphane was not active (Table 1, entry 7), a change to the smaller and more nucleophilic tri-*n*-butylphosphane, a widely used nucleophilic catalyst [39–42], produced coumarin in rather high yields. Concentrating on *n*-Bu_3_P as a very successful catalyst, a solvent screen was performed at the lower temperature of 60 °C (Table 2).

**Table 2:** Screening of solvents in catalyzed coumarin synthesis.^a^

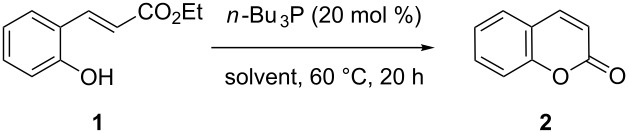			

Entry	Solvent	Time (h)	Yield (%)^b^

1	toluene	28	26
2	isopropanol	28	19
4	EtCN	28	8
5	dioxane	28	2
5	hexane	20	7
6	CHCl_3_	28	21
7	MeOH	22	77
8	MeOH	22	47^c^
9	glycol	22	3
10	glycerol	16	27
11	MeOH/H_2_O (1:1)	16	30
12	CF_3_CH_2_OH	16	43
13	MeCN	16	6
14	DMSO	16	–
15	*tert*-butanol	23	36

^a^Reactions were performed on a 0.5 mmol scale in 1 mL of solvent. ^b^Screening yields were determined by GC and/or HPLC analysis with *N*-pivaloylaniline as the internal standard. ^c^PCy_3_ was used as the catalyst instead of *n*-Bu_3_P.

Conversion to the product in toluene was now considerably reduced (Table 2, entry 1). The broad solvent screening implied that some protic solvents, in particular methanol (Table 2, entry 7), were superior to toluene or polar aprotic solvents. The alternative phosphane PCy_3_ was also briefly tested in that solvent, but the reduced activity implied that there was a negative steric influence with this catalyst (Table 2, entry 8).The scope and utility of the optimal catalyst system *n*-Bu_3_P in MeOH was next studied in preparative reactions with a range of substituted 2’-hydroxycinnamic esters **3**, which were readily obtained by reaction of substituted salicylaldehydes with methyl triphenylphosphoranylidene acetate (Ph_3_P=CHCO_2_Me) [16,28]. They were cyclized to the corresponding substituted coumarins **4** under the optimized conditions, involving 20 mol % of *n*-Bu_3_P as the catalyst in methanol solution at 70 °C (Table 3).

**Table 3:** Substrate scope of the phosphane catalyzed coumarin synthesis.^a^

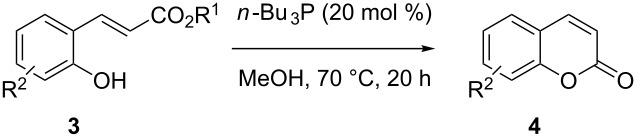			

Entry	Substrate	Product	Yield (%)^b^

1	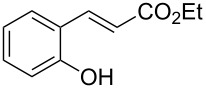 **1**	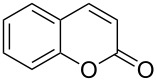 **2**	82
2	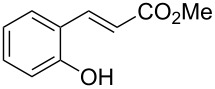 **3a**	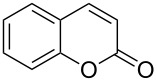 **2**	80
3	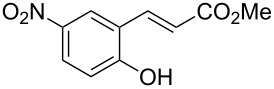 **3b**	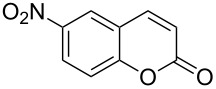 **4b**	–^c^
4	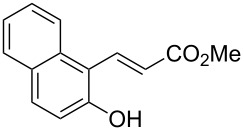 **3c**	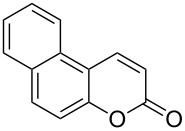 **4c**	96
5	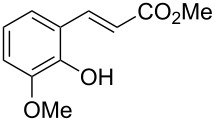 **3d**	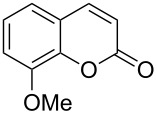 **4d**	88^d^
6	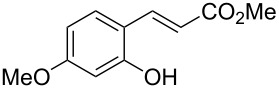 **3e**	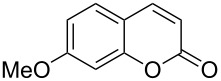 **4e**	83
7	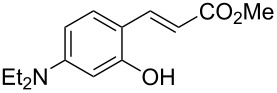 **3f**	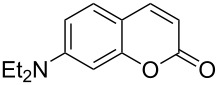 **4f**	96
8	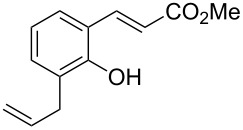 **3g**	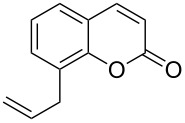 **4g**	96
9	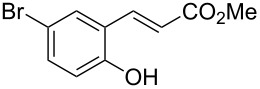 **3h**	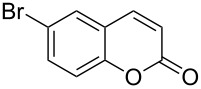 **4h**	75
10	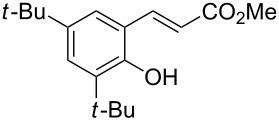 **3i**	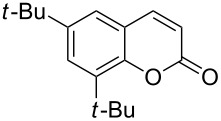 **4i**	99

^a^Reactions were performed on a 1 mmol scale in 1 mL of methanol at 70 °C for 20 h. 15 min prior to workup, the mixture was quenched by addition of 1,2-dibromoethane (20 μL, 0.23 equiv). ^b^Yields of pure isolated material. ^c^No reaction to coumarin observed; starting material (60%) was reisolated. ^d^Reaction performed at 90 °C in a closed vessel.

In these preparative experiments, the reaction mixtures were quenched by the addition of a cocatalytic amount of 1,2-dibromoethane prior to work-up, as described below (see the experimental section). The coumarins were generally obtained in good to excellent yields under the satisfactorily mild conditions of this catalysis. An exception was methyl 4’-nitro-2’-hydroxycinnamate, which did not cyclize to coumarin under the present conditions (Table 3, entry 3). Several of the products in Table 3, including the *N*,*N*-diethylamino- (Table 3, entry 7), and methoxy-derivatives (Table 3, entries 5 and 6) show strong fluorescence.

**Scheme 3:**
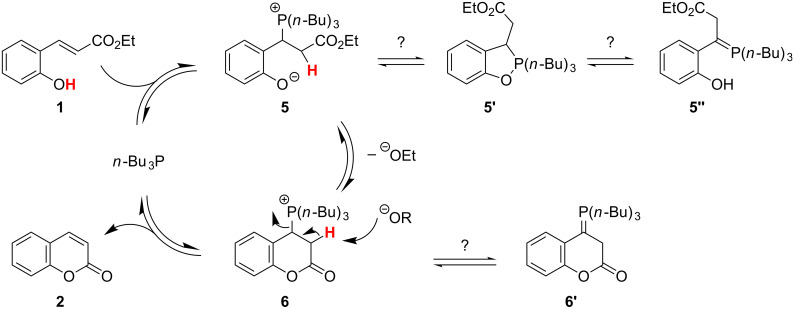
Proposed catalytic cycle, based on ^31^P NMR spectroscopic and color evidence.

The cyclization of (*E*)-2’-hydroxycinnamates **1** and **3** to coumarins **2** and **4** is slow because it requires prior inversion of the double-bond geometry, a process that calls for harsh conditions such as high temperatures (140–250 °C). We have considered a strategy to perform a fast catalytic conjugate addition of a heteronucleophile to the starting material in order to speed up the overall cyclization reaction. Initial experiments with neutral thiols as nucleophiles were not successful. On the other hand, addition of a nucleophilic trialkylphosphane to (*E*)-ethyl 2’-hydroxycinnamate (**1**) in methanol solution, immediately produced a yellow color indicative of the generation of a phenolate anion. When this experiment was carried out in an NMR tube in [*D*_4_]-methanol, ^31^P NMR spectroscopy indicated that *n*-Bu_3_P (δ = −30 ppm) was indeed consumed by a conjugate addition to **1**, with generation of a tributylphosphonium salt (δ = +37 ppm) of the probable zwitterionic structure **5** (Scheme 3; for the ^31^P NMR spectrum see the Supporting Information File 1).

Similar phosphonium phenolates are known to exist either as the zwitterionic (analogue to **5**) or neutral phosphorane structure (analogue to **5’**) [43–44]. Under the conditions of the catalytic reaction, phosphonium (+37 ppm) was the only species of importance and thus represents the resting state of the catalytic reaction, which could be either **5** or **6**. We did not observe a ^31^P NMR signal for *n*-Bu_3_P, or a signal for the phosphorane structure **5’**, which is not favored in the highly polar solvent methanol [43]. Interestingly, we noted that quenching of the catalytic reaction mixtures with a cocatalytic amount of 1,2-dibromoethane prior to work-up had a favorable effect on product yield and purity: First, any of the catalyst *n*-Bu_3_P present will be converted to a water-soluble phosphonium salt, which is easier to separate from the coumarin product than the neutral phosphane is. Second, the yields of coumarins were higher when the quenching procedure was used. This implies that part of the coumarin product may remain associated with the catalyst after full conversion of the starting material, for example in the form of **6** or **6’**. Addition of 1,2-dibromoethane will assist in shifting the equilibrium away from **6**/**6’** to release product **2** and *n*-Bu_3_P, since the latter should react irreversibly with the alkylating reagent.

## Conclusion

In conclusion, we have developed a new catalytic cyclization of (*E*)-alkyl 2’-hydroxycinnamates to coumarins with the aid of a nucleophilic alkyl phosphane catalyst, which allows this conversion to be carried out under much milder conditions compared to established procedures [16,19–29]. The reaction should be of particular utility in the generation of coumarin derivatives in the late stages of multistep syntheses of sensitive targets. This work also illustrates the utility of using organocatalytic rationales (mechanistic predictions) for developing new solutions to synthetic problems.

## Experimental

### General procedure for coumarin synthesis

The starting hydroxycinnamate ester (1 mmol) was inserted in a headspace vial containing a magnetic stirring bar. The vial was flushed with argon and capped. Methanol (1 mL, degassed with argon) was added by syringe through the cap. After addition of tri-*n*-butylphosphane (50 μL, 0.20 mmol; 20 mol %) with a microliter syringe, the solution turned bright yellow. The reaction mixture was heated to 70 °C and stirred for 20 h. The reaction was quenched by the addition of 1,2-dibromoethane (20 μL, 0.23 mmol, 0.23 equiv) and cooled to room temperature. After evaporation, the crude mixture was purified by column chromatography.

#### Reaction example: 7-(*N*,*N*-Diethylamino)coumarin (**4f**)

Prepared according to the general procedure from (*E*)-methyl 3-(2-hydroxy-4-*N,N*-diethylaminophenyl)propenoate (**3f**, 249 mg, 1 mmol) with *n*-Bu_3_P (50 μL, 0.20 mmol, 20 mol %) in MeOH (1 mL). After work-up and column chromatography (EtOAc/hexanes 1:5 + 5% NEt_3_), the product was obtained as a yellow crystalline solid (209 mg, 96%). CAS-Nr. 20571-42-0; mp 90 °C; *R*_f_ = 0.32 (Hex/EtOAc 5:1 + 5% NEt_3_); ^1^H NMR (360 MHz, CDCl_3_) δ 7.54 (d, *J* = 9.4 Hz, 1H), 7.25 (d, *J* = 8.5 Hz, 1H), 6.60 (dd, *J* = 8.6, 2.6 Hz, 1H), 6.51 (d, *J* = 2.6 Hz, 1H), 6.04 (d, *J* = 9.3 Hz, 1H), 3.42 (q, *J* = 7.1 Hz, 4H), 1.21 (t, *J* = 7.1 Hz, 1H) ppm; ^13^C NMR (90 MHz, CDCl_3_) δ 162.2 (C=O), 156.7 (C), 150.5 (C), 143.7 (CH), 128.7 (CH), 109.3 (CH), 108.9 (CH), 108.5 (C), 97.7 (CH), 45.0 (2 × CH_2_), 12.4 (2 × CH_3_) ppm.

See Supporting Information File 1 for experimental details and NMR spectra of coumarins and the precursor 2’-hydroxycinnamates.

## Supporting Information

File 1 Experimental procedures, characterization data and copies of NMR spectra.
