# Supplementary material for: Cyclization of ortho-hydroxycinnamates to coumarins under mild conditions: A nucleophilic organocatalysis approach
Source: Beilstein J Org Chem. 2012 Sep 26;8:1630–6. doi: 10.3762/bjoc.8.186 (PMC3510995; doi:10.3762/bjoc.8.186)

Supporting Information

for

Cyclization of *ortho*-hydroxycinnamates to coumarins under mild conditions: A nucleophilic organocatalysis approach

Florian Boeck, Max Blazejak, Markus R. Anneser and Lukas Hintermann*

Address: Department Chemie, Technische Universität München, Lichtenbergstr. 4, 85748 Garching, Germany

Email: Lukas Hintermann* - lukas.hintermann@tum.de

* Corresponding author

Experimental procedures, characterization data and copies of NMR spectra

Contents

1. General S2

2. Synthesis of methyl 2'-hydroxycinnamates 3 S2

3. Synthesis of coumarins S4

4. ^{31}P NMR spectra of reaction mixture S7

5. NMR spectra S8

1. General

Reactions were carried out under an argon atmosphere. F-TEDA and NCS were obtained from Aldrich. Column chromatography was performed by using silica gel 60 (particle size 40–63 μm). NMR spectra were recorded in $CDCl_3$ at ambient temperature (20–25 °C) relative to TMS, unless otherwise noted. ^{13}C NMR shifts are relative to TMS, but referenced through the solvent peaks. Abbreviations: aq = dissolved in water. CC is column chromatography (on SiO_2). sat = "saturated solution of". THF is tetrahydrofuran.

2. Synthesis of methyl 2'-hydroxycinnamates 3

General procedure for the synthesis of methyl 2'-hydroxycinnamates 3 (GP1)

Methyl(triphenylphosphoranylidene)acetate (Ph_3P=$CHCO_2Me$; 1.5 equiv) was added under stirring to a solution of 2-hydroxyarylaldehyde (1.0 equiv) in dichloromethane (5 mL/mmol) at 0 °C, and the resulting yellow solution was stirred overnight (15 h) with warming to rt. After the addition of SiO_2, the solvent was removed under reduced pressure to give a fine powdery residue. This material was placed on top of a short silica gel column and eluted with EtOAc/hexanes 1:1. The combined product fractions were evaporated to a small volume and the product was crystallized by covering the concentrated solution with hexanes and allowing it to stand in a fridge (4 °C).

**Product**	**Scale**	**Yield**
(*E*)-Methyl 2'-hydroxycinnamate (**3a**)	14 mmol	91%
(*E*)-Methyl 2'-hydroxy-5'-nitrocinnamate (**3b**)	11 mmol	15%
(*E*)-Methyl 3-(2-hydroxynaphthalen-1-yl)acrylate (**3c**)	12.4 mmol	88%
(*E*)-Methyl 2'-hydroxy-3'-methoxycinnamate (**3d**)	5 mmol	85%
(*E*)-Methyl 2'-hydroxy-4'-methoxycinnamate (**3e**)	6.6 mmol	93%
(*E*)-Methyl 4'-*N*,*N*-diethylamino-2'-hydroxycinnamate (**3f**)	15 mmol	70%
(*E*)-Methyl 3'-allyl-2'-hydroxycinnamate (**3g**)	2 mmol	96%
(*E*)-Methyl 5'-bromo-2'-hydroxycinnamate (**3h**)	15 mmol	82%
(*E*)-Methyl 3',5'-di-*tert*-butyl-2'-hydroxycinnamate (**3i**)	8.5 mmol	91%
(*E*)-Methyl 3',5'-dichloro-2'-hydroxycinnamate	10.5 mmol	99%

Specific substances

Most of the 2'-hydroxycinnamates were known compounds and their data corresponded to those described in the literature. The following compounds or data have not been described in the literature:

(*E*)-Methyl 2'-hydroxy-5'-nitrocinnamate (3b)

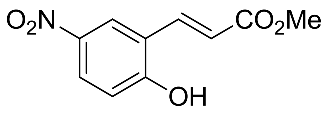

Synthesized according to the general procedure GP1, yield 15%, not optimized; the product was difficult to separate from side-products.

^{1}H NMR (360 MHz, $CDCl_3$): δ = 7.69 (d, *J* = 9.6 Hz, 1H), 7.21 (t, *J* = 8.0 Hz, 1H), 7.10–7.05 (2H), 6.43 (d, *J* = 9.5 Hz, 1H), 3.97 (s, 3H) ppm. ^{13}C NMR (90 MHz, $CDCl_3$): δ = 160.2 (C=O), 147.3 (C), 143.8 (C), 143.5 (CH), 124.3 (CH), 119.5 (C), 119.3 (CH), 117.0 (CH), 113.8 (CH), 56.3 (CH_3) ppm.

(*E*)-Methyl 2'-hydroxy-3'-methoxycinnamate (3d)

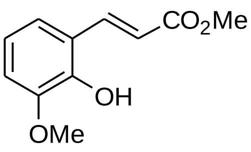

Synthesized according to the general procedure GP1, 85% yield. CAS-Nr. 1135-24-6. Mp 114 °C. ^{1}H NMR (360 MHz, $CDCl_3$): δ = 7.95 (d, *J* = 16.1 Hz, 1H), 7.08 (dd, *J* = 6.8, 1.6 Hz, 1H), 6.88–6.84 (m, 2H), 6.61 (d, *J* = 16.1 Hz, 1H), 6.17 (s, 1H), 3.91 (s, 3H), 3.80 (s, 3H) ppm. ^{13}C NMR (90 MHz, $CDCl_3$): δ = 167.9 (C=O), 146.8 (C), 145.3 (C), 139.8 (CH), 120.9 (CH), 120.8 (C), 119.7 (CH), 118.8 (CH), 111.7 (CH), 56.2 (CH_3), 51.6 (CH_3) ppm.

(*E*)-Methyl 3'-allyl-2'-hydroxycinnamate (3g)

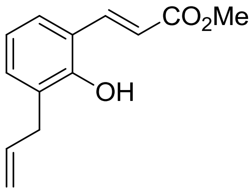

Synthesized according to the general procedure GP1, 96% yield.

Mp 77 °C. ^{1}H NMR (360 MHz, $CDCl_3$): δ = 8.02 (d, *J* = 16.1 Hz, 1H), 7.40 (dd, *J* = 7.8, 1.5 Hz, 1H), 7.14 (dd, *J* = 7.5, 1.5, 1H), 6.90 (t, *J* = 7.6 Hz, 1H), 6.53 (d, *J* = 16.1 Hz, 1H), 6.07–5.96 (m, 1H), 5.28–5.20 (m, 2H), 3.80 (s, 3H), 3.45 (t, *J* = 6.2 Hz, 2H) ppm. ^{13}C NMR (90 MHz, $CDCl_3$): δ = 167.9 (C=O), 153.8 (C), 140.0 (CH), 137.7 (CH), 132.4 (CH), 127.4 (CH), 125.4 (C), 122.4 (C), 120.9 (CH), 118.5 (CH), 117.7 (CH), 51.6 (CH_3), 25.7 (CH_2) ppm.

3. Synthesis of coumarins

General procedure for synthesis of coumarins (GP2):

The starting alkyl hydroxycinnamate (1 mmol) was inserted in a headspace vial with a magnetic stirring bar. The vial was flushed with argon and capped. Methanol (1 mL, degassed with argon) was added by syringe through the cap. After addition of tri-*n*-butylphosphane (50 μL, 0.2 mmol; 20 mol %) with a microliter syringe, the solution turned bright yellow. The reaction mixture was heated to 70 °C and stirred for 20 h. The reaction was quenched by the addition of 1,2-dibromoethane (20 μL, 0.23 mmol, 0.23 equiv) and cooled to room temperature. After evaporation, the crude mixture was purified by column chromatography.

Substances

2*H*-Chromen-2-one (coumarin; 2 = 4a)

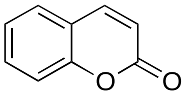

Synthesized according to the general procedure from (*E*)-ethyl 2'-hydroxy-cinnamate (192 mg, 1 mmol). After work-up and CC (SiO_2, EtOAc/hexanes 1:5 + 5% NEt_3), a colorless crystalline solid (120 mg, 82 % yield) was isolated.

CAS-Nr. 91-64-5. Mp 70–70.5 °C. R_f = 0.28 (EtOAc/hexanes 1:5 + 5% NEt_3). ^1H NMR (360 MHz, $CDCl_3$): δ = 7.71 (dd, *J* = 9.5, 0.5 Hz, 1H), 7.56–7.51 (m, 1H), 7.49 (dd, *J* = 7.7, 1.6 Hz, 1H), 7.34 (d, *J* = 8.1 Hz, 1H), 7.27 (dt, *J* = 7.6, 1.1 Hz, 1H), 6.43 (d, *J* = 8.5 Hz, 1H) ppm. ^{13}C NMR (90 MHz, $CDCl_3$): δ = 160.1 (C=O), 154.0 (C), 143.3 (CH), 131.8 (CH), 127.8 (CH), 124.3 (CH), 118.8 (C), 116.8 (CH), 116.6 (CH) ppm.

3*H*-Benzo[*f*]chromen-3-one (4c)

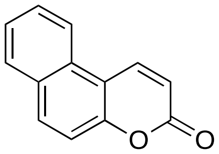

Synthesized according to the general procedure from (*E*)-methyl 3-(2-hydroxynaphthalen-1-yl)acrylate (228 mg, 1 mmol). After work-up and CC (EtOAc/hexanes 1:10 + 5% NEt_3) a yellowish crystalline solid (189 mg, 96%) was obtained.

CAS-Nr.: 4352-89-0. Mp 118 °C. R_f = 0.32 (EtOAc/hexanes 1:10 + 5% NEt_3). ^1H NMR (360 MHz, $CDCl_3$): δ = 8.47 (d, *J* = 9.7 Hz, 1H), 8.21 (d, *J* = 8.4 Hz, 1H), 7.97 (d, *J* = 9.0 Hz, 1H), 7.90 (d, *J* = 8.1 Hz, 1H), 7.71–7.66 (m, 1H), 7.59–7.54 (m, 1H), 7.44 (d, *J* = 9.2 Hz, 1H), 6.56 (d, *J* = 9.8 Hz, 1H) ppm. ^{13}C NMR (90 MHz, $CDCl_3$): δ = 160.9 (C=O), 153.9 (C), 139.1

(CH), 133.1 (CH), 130.3 (CH), 129.0 (C), 128.3 (CH), 126.1 (CH), 121.3 (CH), 117.1 (CH), 115.6 (CH), 113.0 (C) ppm.

8-Methoxy-2*H*-chromen-2-one (8-methoxycoumarin, 4d)

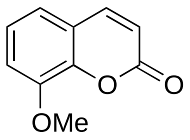

Synthesized according to the general procedure from (*E*)-methyl 2-hydroxy-3-methoxycinnamate (208 mg, 1 mmol). After work-up and CC (EtOAc/hexanes 1:5 + 5% NEt_3) a colorless crystalline solid (155 mg, 88%) was isolated.

CAS-Nr. 2445-81-0. Mp 91 °C; R_f = 0.18 (EtOAc/hexanes 1:5 + 5% NEt_3). ^{1}H NMR (360 MHz, $CDCl_3$): δ = 7.69 (d, *J* = 9.6 Hz, 1H), 7.21 (t, *J* = 7.8 Hz, 1H), 7.11–7.05 (m, 1H), 6.44 (d, *J* = 9.5 Hz, 1H), 3.97 (s, 3H) ppm. ^{13}C NMR (90 MHz, $CDCl_3$): δ = 180.2 (C=O), 147.3 (C), 143.8 (C), 143.6 (CH), 124.3 (CH), 119.5 (C), 119.3 (CH), 117.0 (CH), 113.8 (CH), 56.8 (CH_3) ppm.

7-Methoxy-2*H*-chromen-2-one (7-methoxycoumarin, 4e)

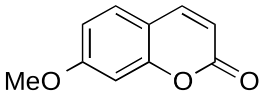

Synthesized according to the general procedure from (*E*)-methyl 2-hydroxy-4-methoxy-cinnamate (228 mg, 1 mmol). After work-up and CC (EtOAc/hexanes 1:5 + 5% NEt_3) a colorless crystalline solid (147 mg, 83%) was obtained.

CAS-Nr. 531-59-9. Mp 119 °C. R_f = 0.27 (EtOAc/hexanes 1:5 + 5% NEt_3). ^{1}H NMR (360 MHz, $CDCl_3$): δ = 7.65 (d, *J* = 9.4 Hz, 1H), 7.38 (d, *J* = 8.5 Hz, 1H), 6.85 (dd, *J* = 8.5, 2.4 Hz, 1H), 6.81 (d, *J* = 2.4 Hz, 1H), 6.25 (d, *J* = 9.5 Hz, 1H), 3.87 (s, 3H) ppm. ^{13}C NMR (90 MHz, $CDCl_3$): δ = 162.8 (C=O), 161.2 (C), 155.9 (C), 143.4 (CH), 128.8 (CH), 128.8 (CH), 113.1 (CH), 112.6 (CH), 112.5 (C), 100.9 (CH), 55.8 (CH_3) ppm.

7-(*N*,*N*-Diethylamino)-2*H*-chromen-2-one (7-diethylaminocoumarin, 4f)

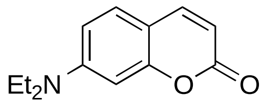

Synthesized according to the general procedure from (*E*)-methyl 2-hydroxy-4'-*N,N*-diethylaminocinnamate (249 mg, 1 mmol). After work-up and CC (EtOAc/hexanes 1:5 + 5% NEt_3) a yellow crystalline solid (209 mg, 96%) was isolated.

CAS-Nr. 20571-42-0. Mp 90 °C. R_f = 0.32 (EtOAc/hexanes 1:5 + 5% NEt_3). ^{1}H NMR (360 MHz, $CDCl_3$): δ = 7.54 (d, *J* = 9.4 Hz, 1H), 7.25 (d, *J* = 8.5 Hz, 1H), 6.60 (dd, *J* = 8.6, 2.6 Hz, 1H), 6.51 (d, *J* = 2.6 Hz, 1H), 6.04 (d, *J* = 9.3 Hz, 1H), 3.42 (q, *J* = 7.1 Hz, 4H), 1.21 (t, *J* = 7.1 Hz, 1H) ppm. ^{13}C NMR (90 MHz, $CDCl_3$): δ = 162.2 (C=O), 156.7 (C), 150.5 (C),

143.7 (CH), 128.7 (CH), 109.3 (CH), 108.9 (CH), 108.5 (C), 97.7 (CH), 45.0 (2 x CH_2), 12.4 (2 x CH_3) ppm.

8-Allyl-2*H*-chromen-2-one (8-(prop-2-en-1-yl)coumarin, 4g)

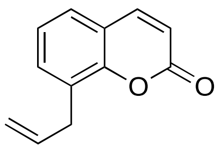

Synthesis according to the general procedure from (*E*)-methyl 2'-hydroxy-3'-(prop-2-en-1-yl)cinnamate (218 mg, 1 mmol). After work-up and CC (EtOAc/hexanes 1:10 + 5% NEt_3) a colorless crystalline solid (178 mg, 96%) was isolated.

CAS-Nr. 176046-05-2. Mp 44 °C. R_f = 0.35 (EtOAc/hexanes 1:10 + 5% NEt_3). ^{1}H NMR (360 MHz, $CDCl_3$): δ = 7.71 (d, J = 9.5 Hz, 1H), 7.41 (d, J = 7.5 Hz, 3H), 7.35 (dd, J = 7.7, 1.7 Hz, 1H), 6.41 (t, J = 7.6 Hz, 1H), 6.07–5.96 (m, 1H), 5.17–5.13 (m, 1H), 5.12–5.10 (m, 1H), 3.62 (d, J = 6.6 Hz, 2H) ppm. ^{13}C NMR (90 MHz, $CDCl_3$): δ = 160.7 (C=O), 151.8 (C), 148.7 (CH), 135.2 (CH), 132.3 (CH), 128.3 (C), 126.0 (CH), 124.1 (CH), 118.7 (C), 116.8 (CH), 116.4 (CH_2), 33.1 (CH) ppm.

6-Bromo-2*H*-chromen-2-one (6-bromocoumarin, 4h)

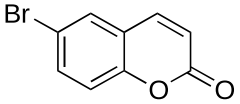

Synthesis according to the general procedure from (*E*)-methyl 2'-hydroxy-5'-bromo-cinnamate (257 mg, 1 mmol). After work-up and CC (EtOAc/hexanes 1:5 + 5% NEt_3) a colorless crystalline solid (169 mg, 75%) was isolated.

CAS-Nr. 19063-55-9. Mp 165 °C. R_f = 0.13 (EtOAc/hexanes 1:5 + 5% NEt_3). ^{1}H NMR (360 MHz, $CDCl_3$): δ = 7.65–7.61 (3H), 7.22 (d, J = 9.5 Hz, 3H), 6.47 (d, J = 9.6 Hz, 1H) ppm. ^{13}C NMR (90 MHz, $CDCl_3$): δ = 159.9 (C=O), 152.9 (C), 142.1 (CH), 134.6 (CH), 130.2 (CH), 120.3 (C), 118.6 (CH), 117.9 (CH), 117.0 (C) ppm.

6,8-Di-*tert*-butyl-2*H*-chromen-2-one (6,8-di-*tert*-butylcoumarin, 4i)

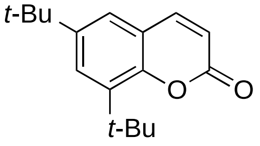

Synthesized according to the general procedure from (*E*)-methyl 2'-hydroxy-3',5'-di-*tert*-butylcinnamate (290 mg, 1 mmol). After work-up and CC (hexanes + 5% NEt_3) a colorless oil (256 mg, 99%) was isolated.

R_f = 0.76 (hexanes + 5% NEt_3). ^{1}H NMR (360 MHz, $CDCl_3$): δ = 7.69 (d, J = 9.5 Hz, 1H), 7.58 (d, J = 2.3 Hz, 1H), 7.30 (d, J = 2.3 Hz, 1H), 6.38 (d, J = 9.5 Hz, 1H), 1.52 (s, 9H), 1.36 (s, 9H) ppm. ^{13}C NMR (90 MHz, $CDCl_3$): δ = 160.9 (C=O), 150.8 (C), 146.7 (C), 144.8 (CH), 137.4 (C), 127.1 (CH), 122.5 (CH), 118.7 (C), 115.5 (CH), 35.1 (CH), 34.7 (C), 31.4 (CH_3), 29.9 (CH_3) ppm.

4. ^{31}P NMR spectra of the reaction mixture

A catalytic reaction was followed by ^{31}P NMR spectroscopy in [D$_4$]-MeOH solution. The reference sample of n-Bu$_3$P in MeOH shows a broad signal at $\delta = -30$ ppm, plus impurities in the 40 to 65 ppm range (other phosphines and oxides) already present in the commercial sample. In the course of the reaction, only a peak at $\delta = +37$ ppm is visible, which may correspond to a phosphonium salt (n-Bu$_3$P$^+$-R) resting state.

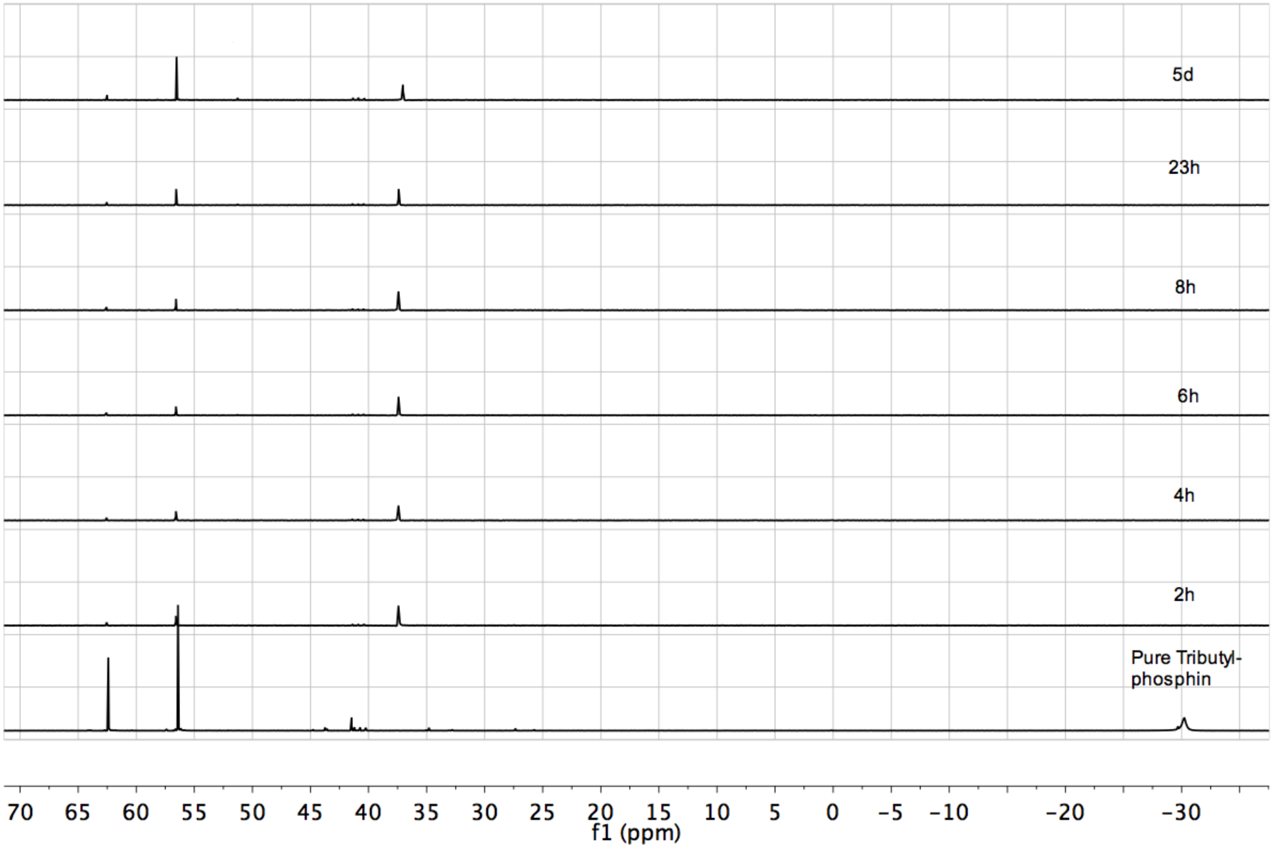

5. NMR spectra

^{1}H NMR (360 MHz) of 5-nitro-2-hydroxy-(*E*)-cinnamic acid methyl ester (3b)

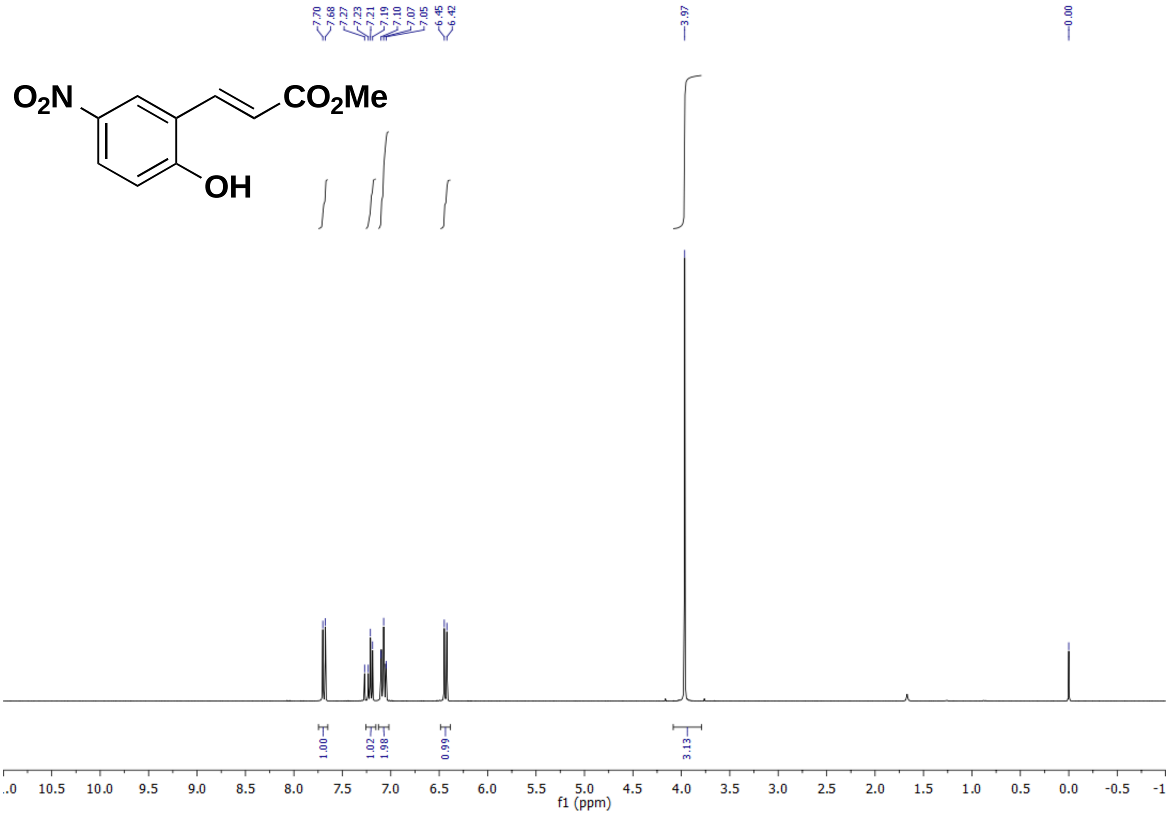

^{13}C NMR (90 MHz) of 5-nitro-2-hydroxy-(*E*)-cinnamic acid methyl ester (3b)

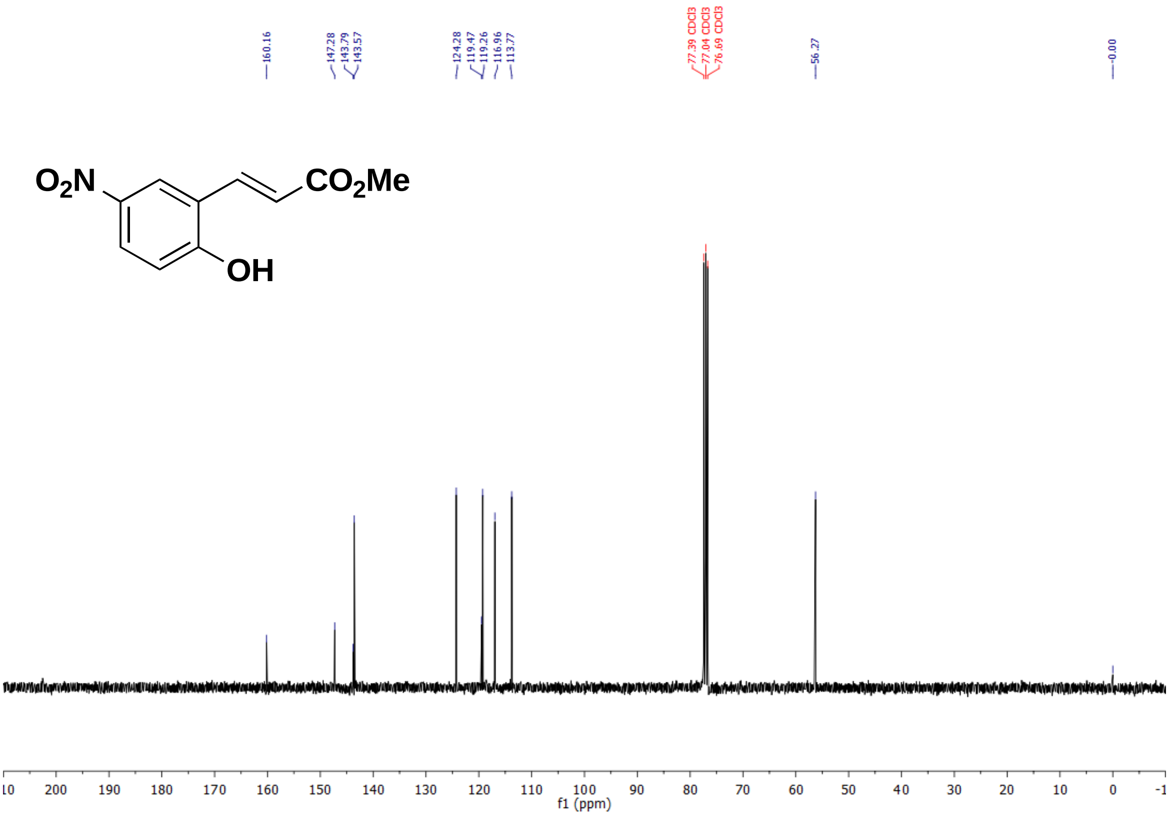

^{1}H NMR (360 MHz) of 2-hydroxy-3-methoxy-(*E*)-cinnamic acid methyl ester (3d)

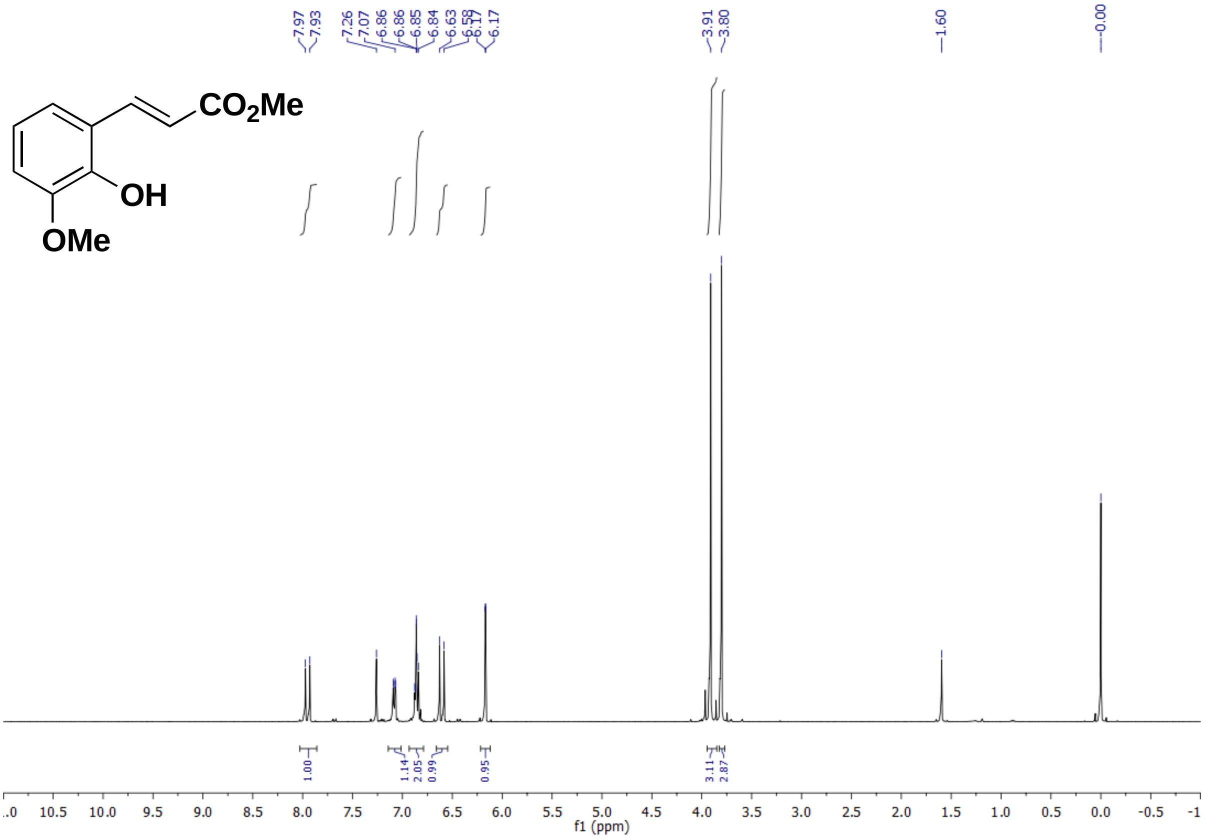

^{13}C NMR (90 MHz) of 2-hydroxy-3-methoxy-(*E*)-cinnamic acid methyl ester (3d)

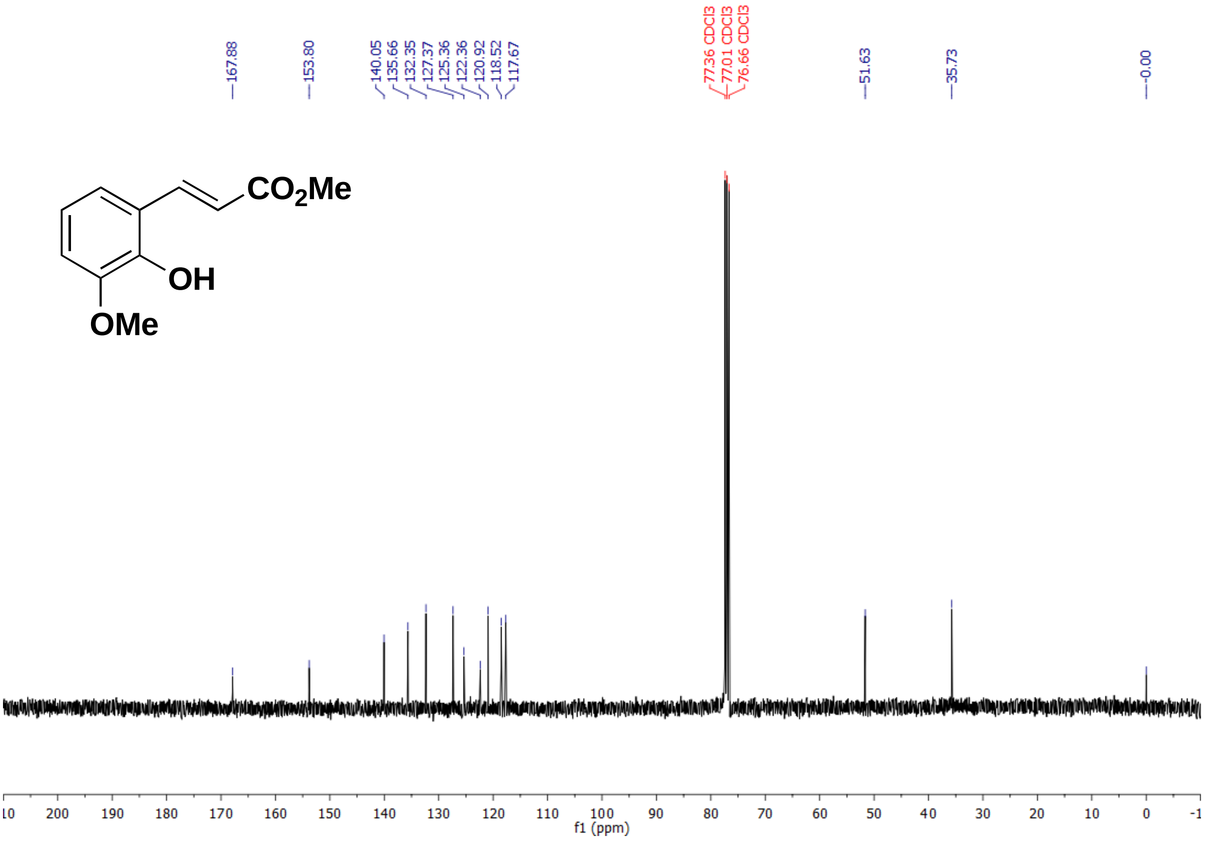

^{1}H NMR (360 MHz) of 3-allyl-2-hydroxy-(*E*)-cinnamic acid methyl ester (3g)

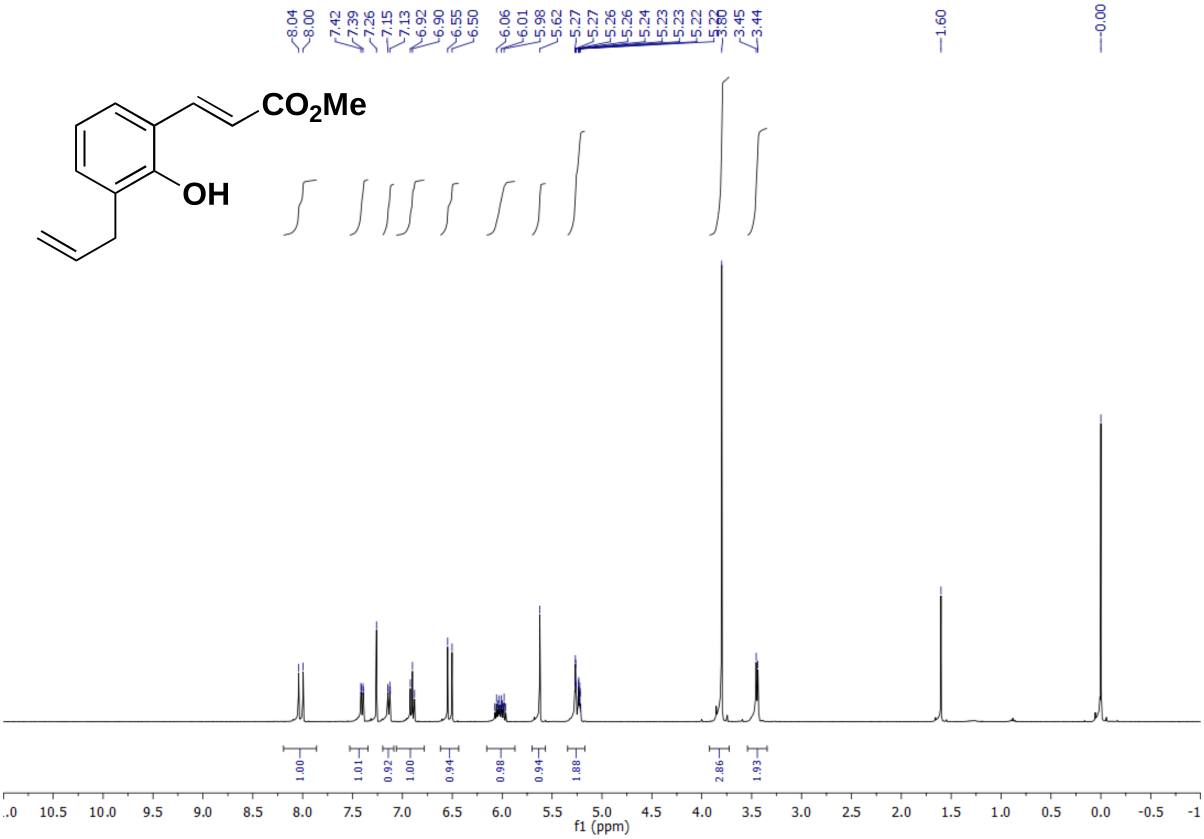

^{13}C NMR (90 MHz) of 3-allyl-2-hydroxy-(*E*)-cinnamic acid methyl ester (3g)

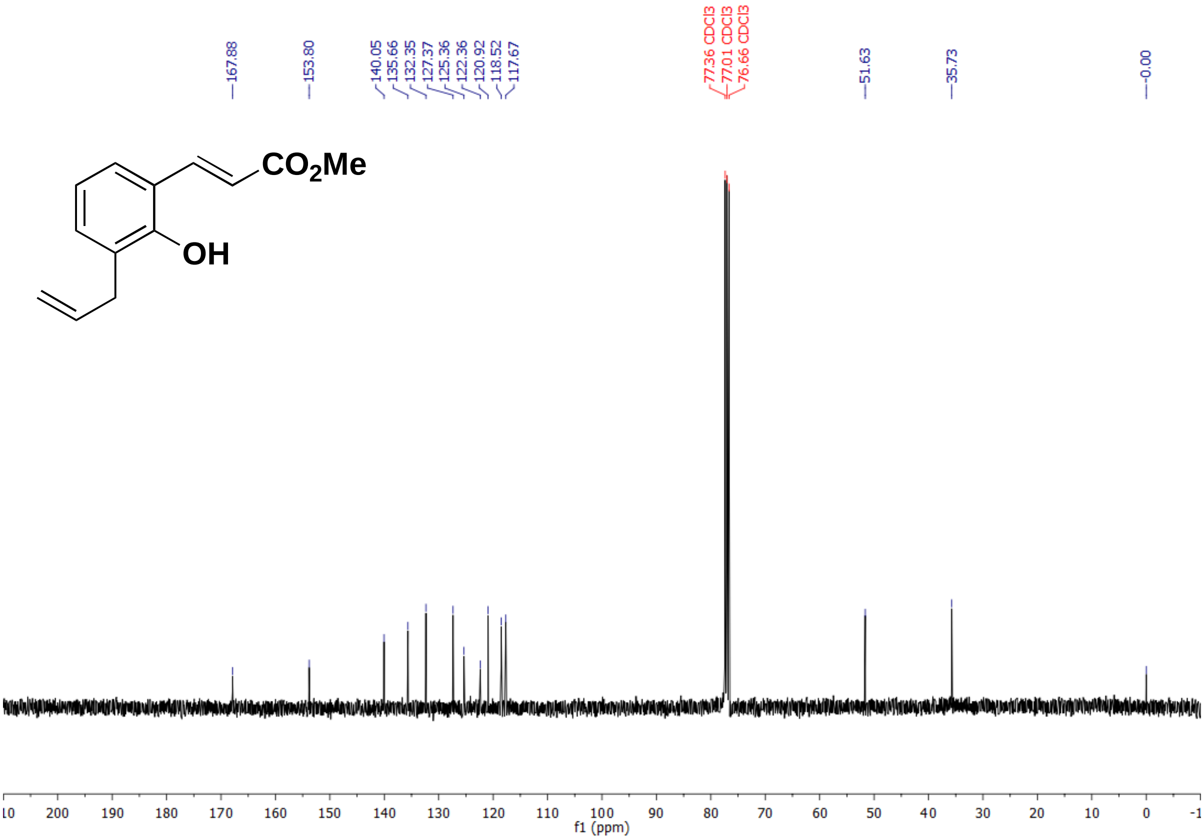

^{1}H NMR (360 MHz, $CDCl_3$) of coumarin (2 = 4a)

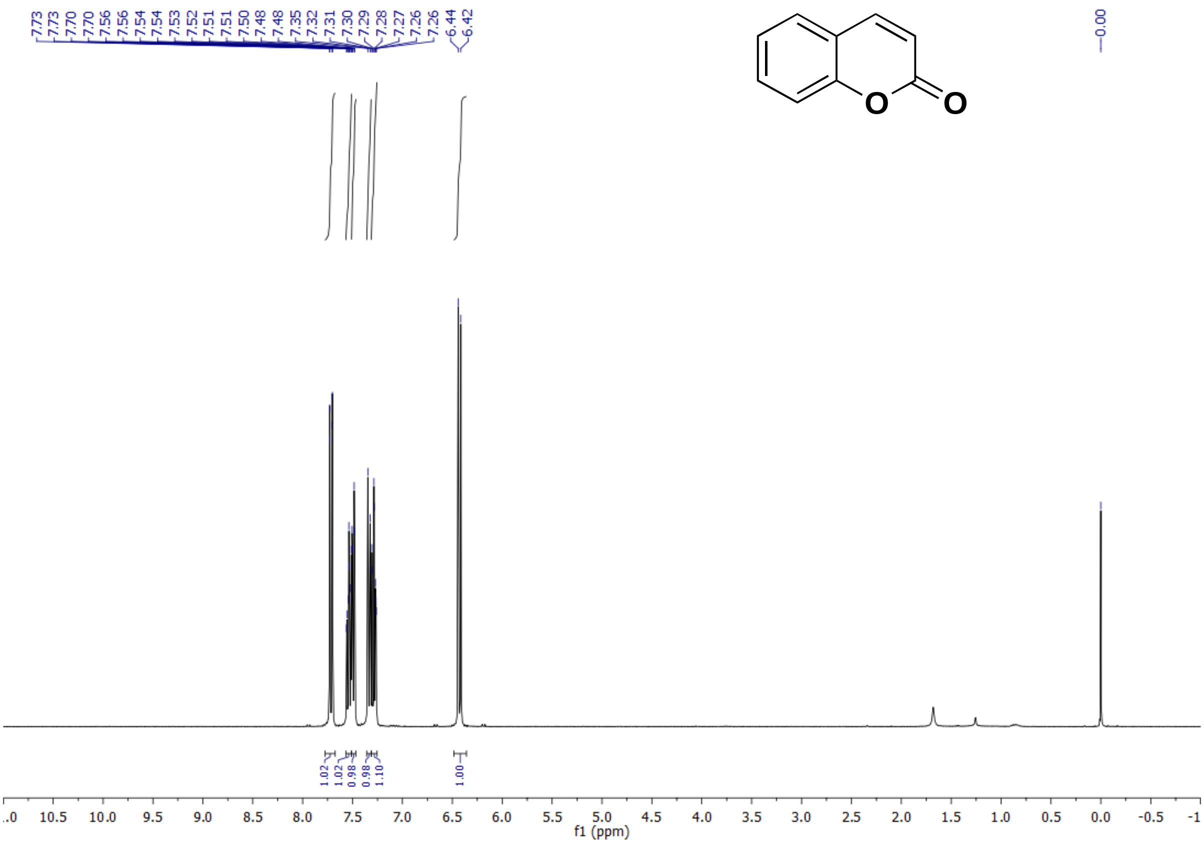

^{13}C NMR (90 MHz, $CDCl_3$) of coumarin (2 = 4a)

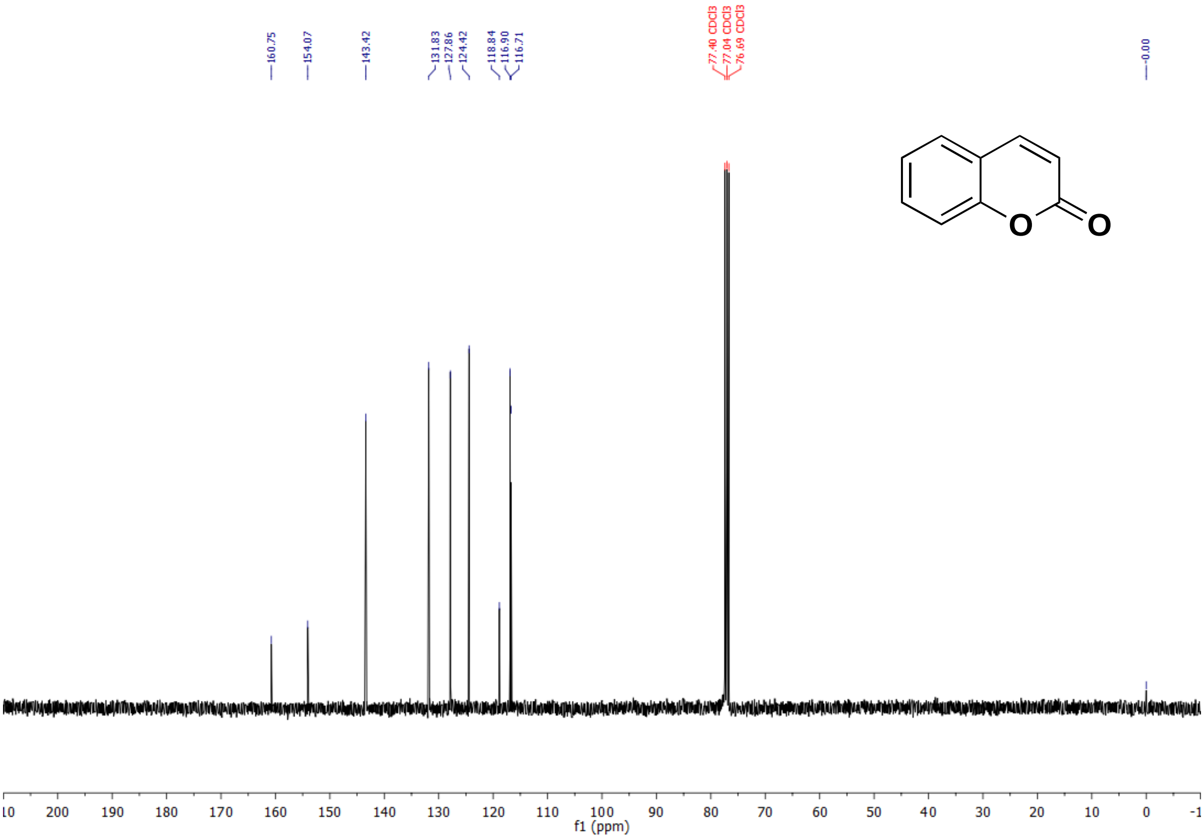

^{1}H NMR (360 MHz, $CDCl_3$) of 3*H*-benzo[*f*]chromen-3-one (4c)

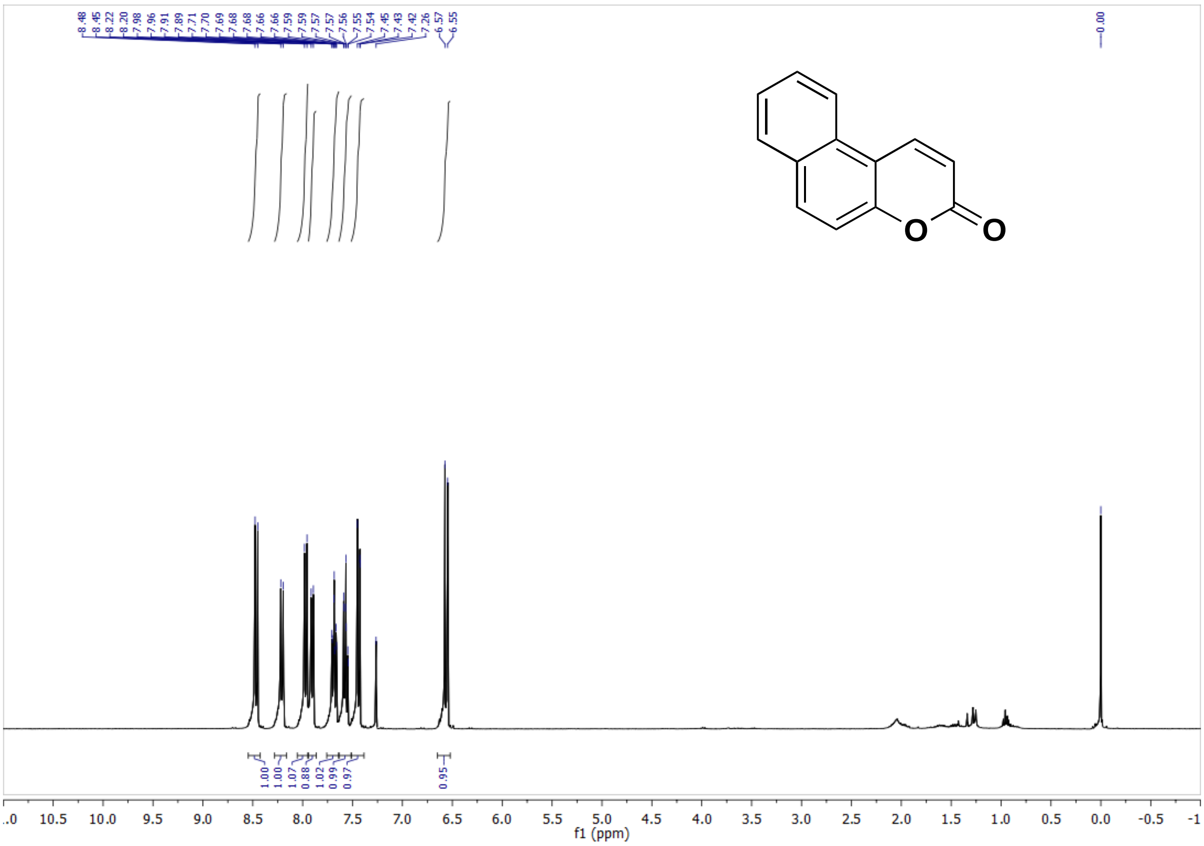

^{13}C NMR (90 MHz, $CDCl_3$) of 3*H*-benzo[*f*]chromen-3-one (4c)

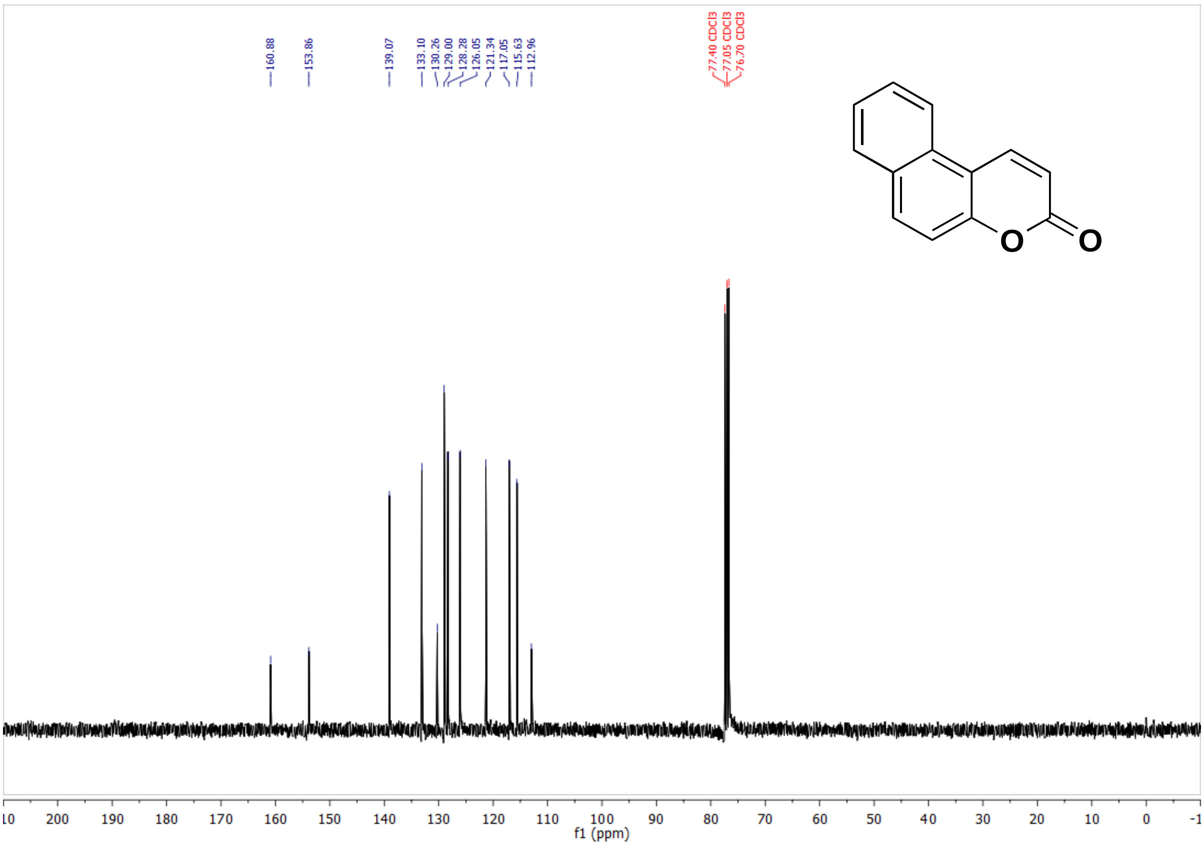

^{1}H NMR (360 MHz, $CDCl_3$) of 8-methoxycoumarin (4d)

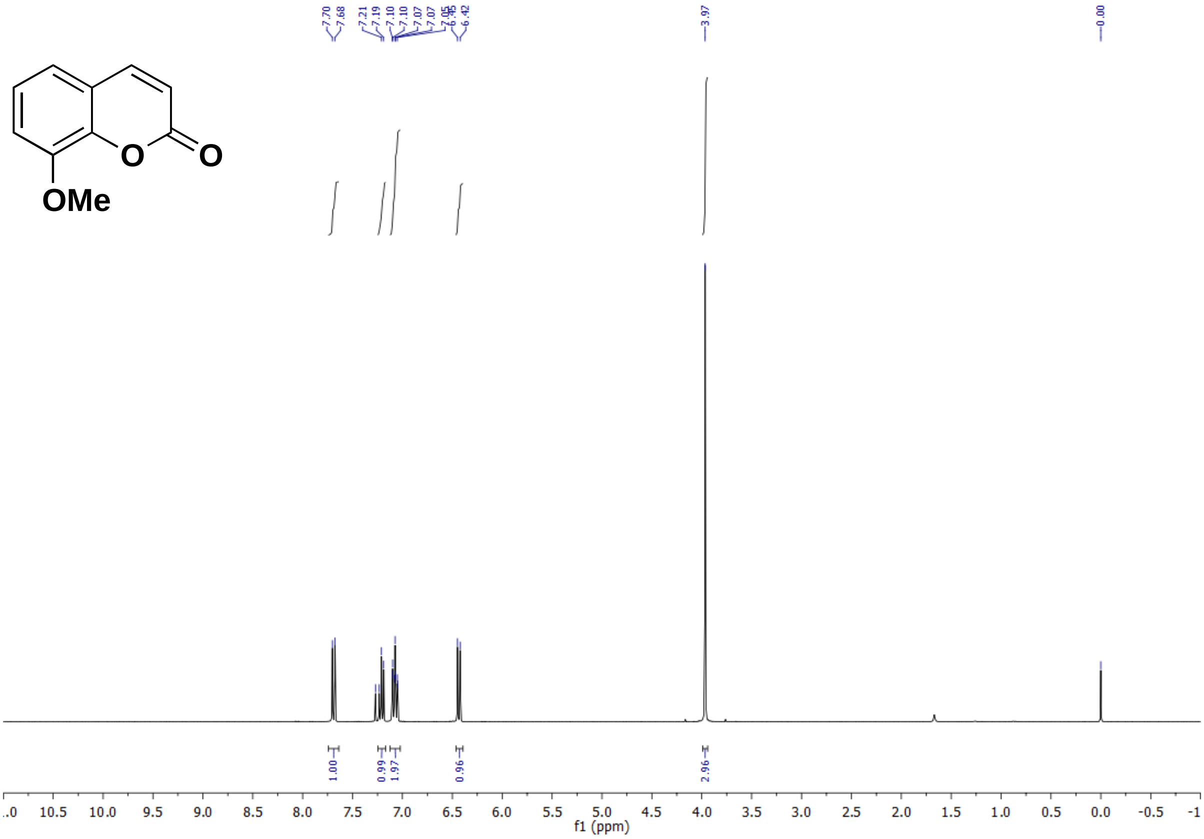

^{13}C NMR (90 MHz, $CDCl_3$) of 8-methoxycoumarin (4d)

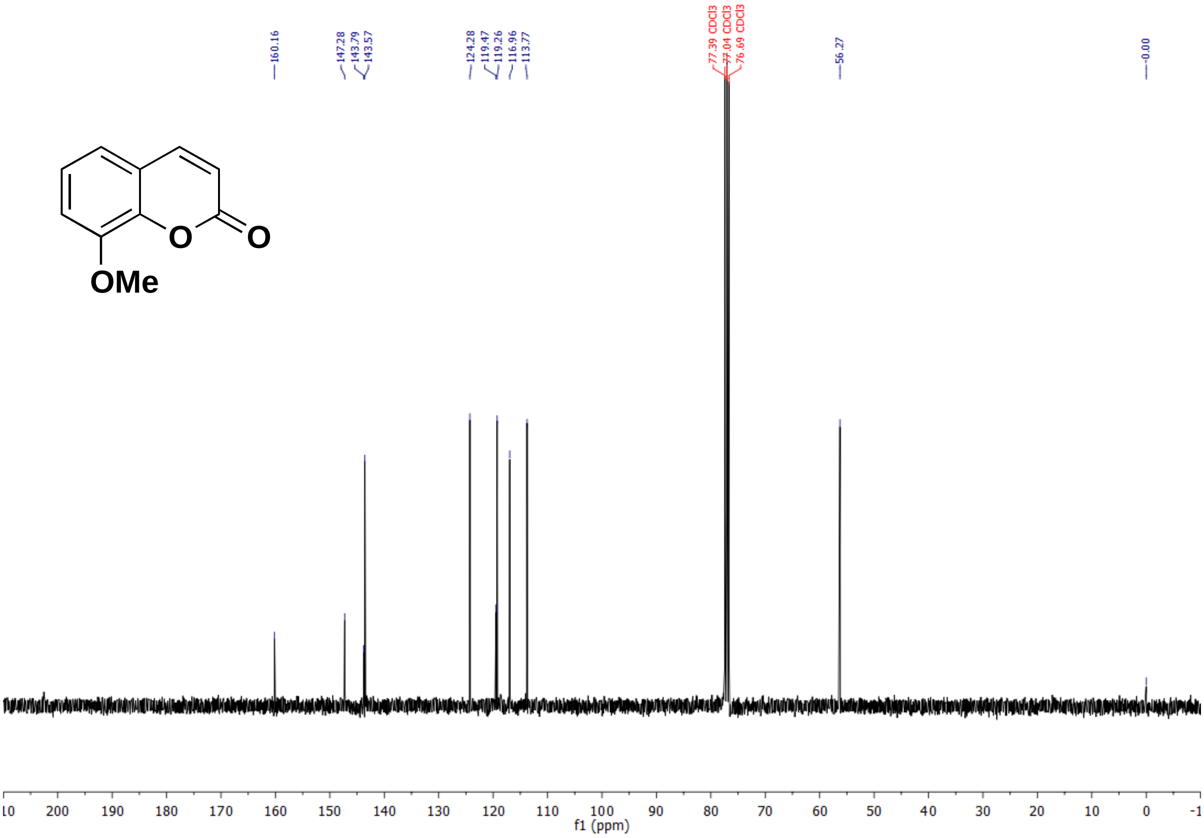

^{1}H NMR (360 MHz, $CDCl_3$) of 7-methoxycoumarin (4e)

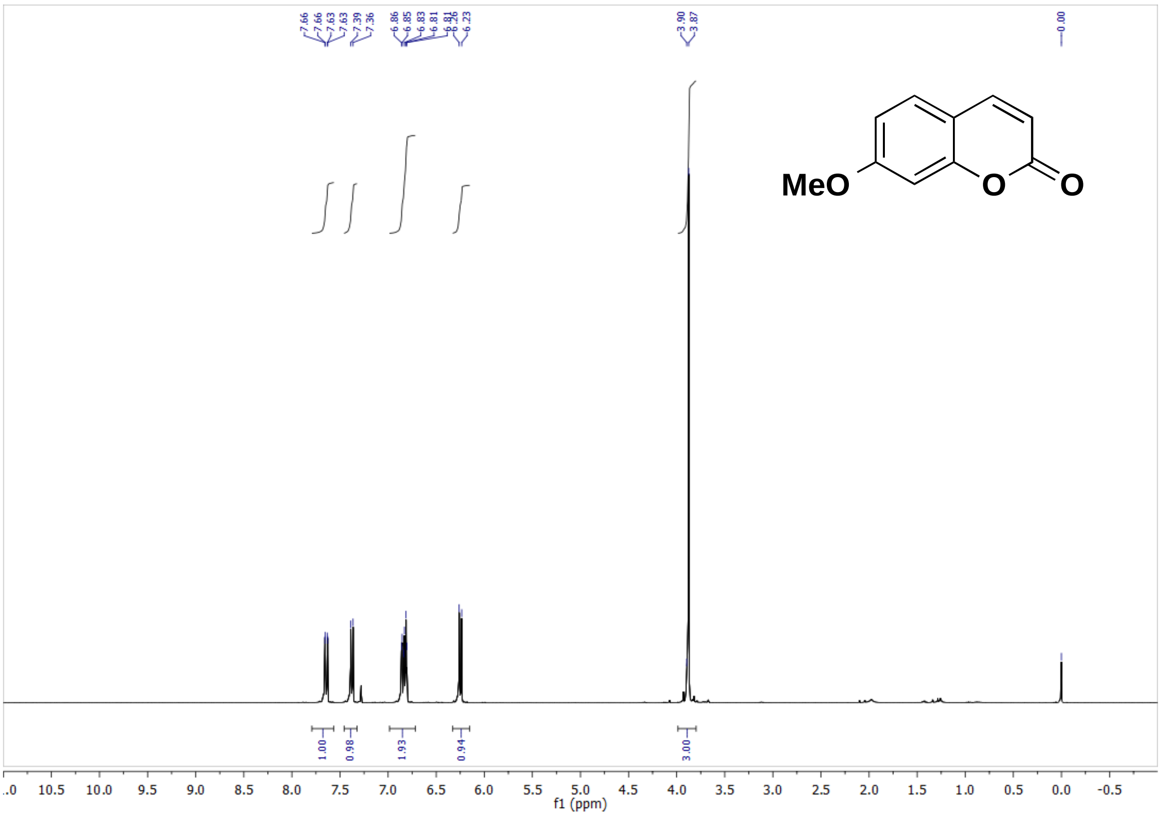

^{13}C NMR (90 MHz, $CDCl_3$) of 7-methoxycoumarin (4e)

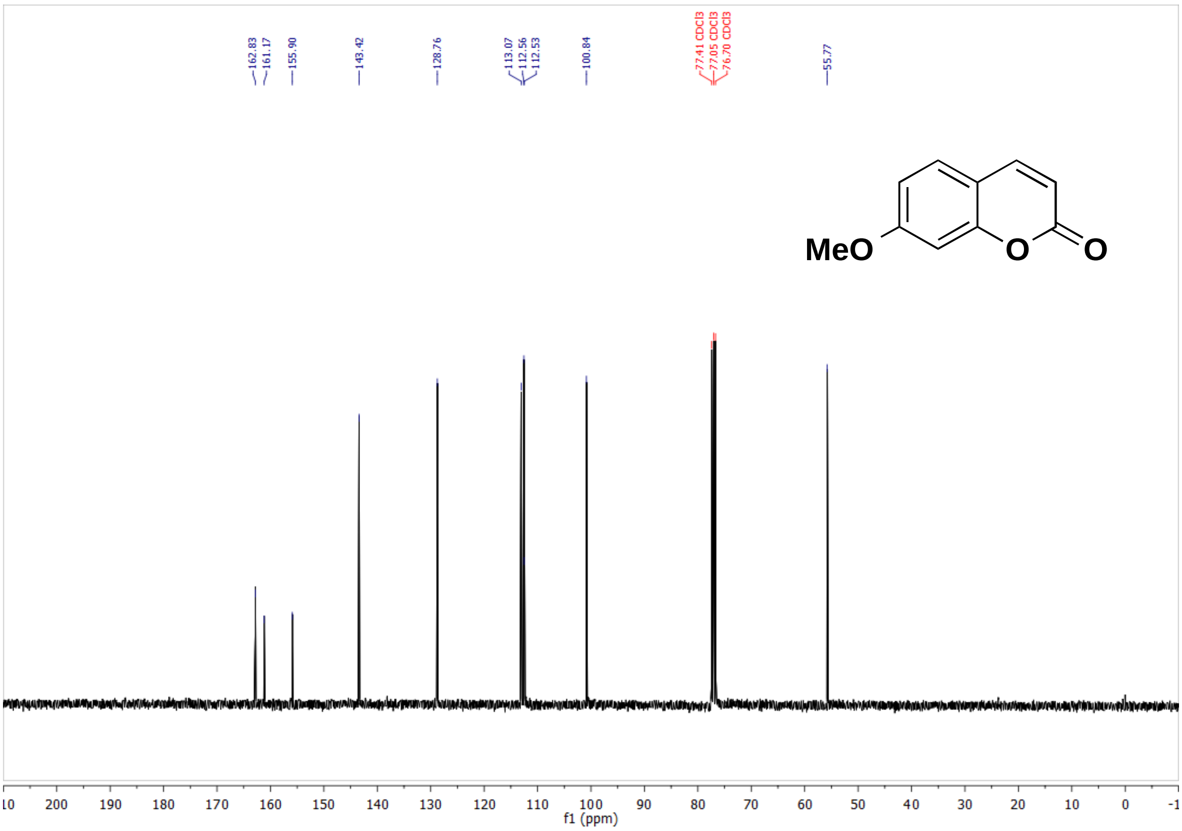

^{1}H NMR (360 MHz, $CDCl_3$) of 7-(diethylamino)coumarin (4f)

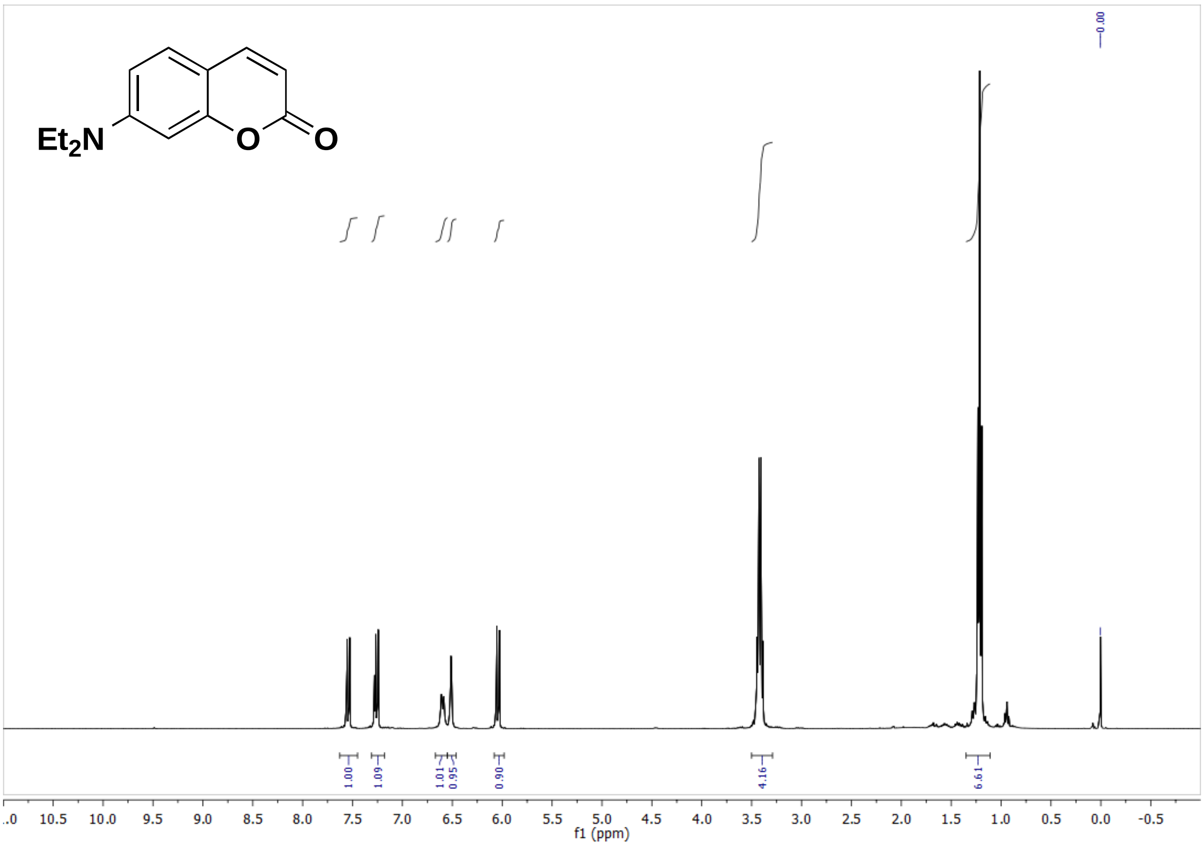

^{13}C NMR (90 MHz, $CDCl_3$) of 7-(diethylamino)coumarin (4f)

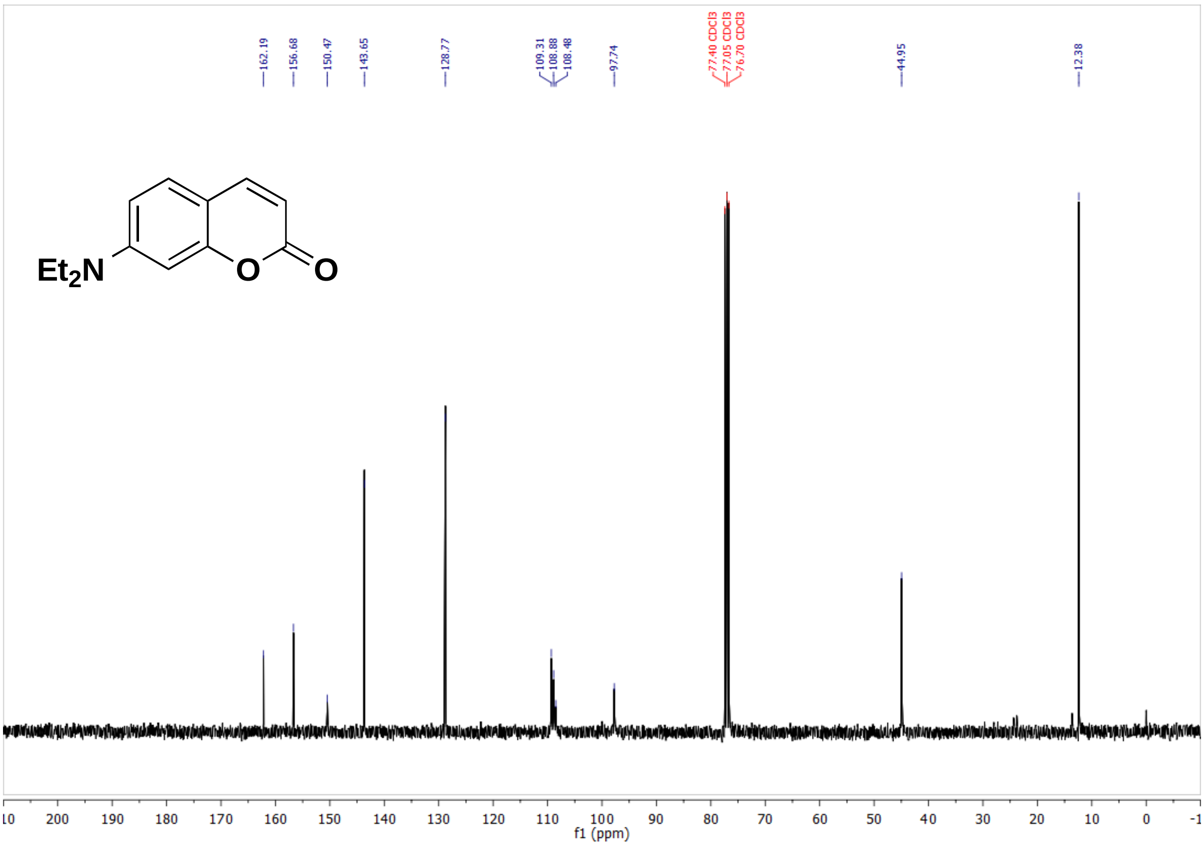

^{1}H NMR (360 MHz, $CDCl_3$) of 8-allyl-2*H*-chromen-2-one (4g)

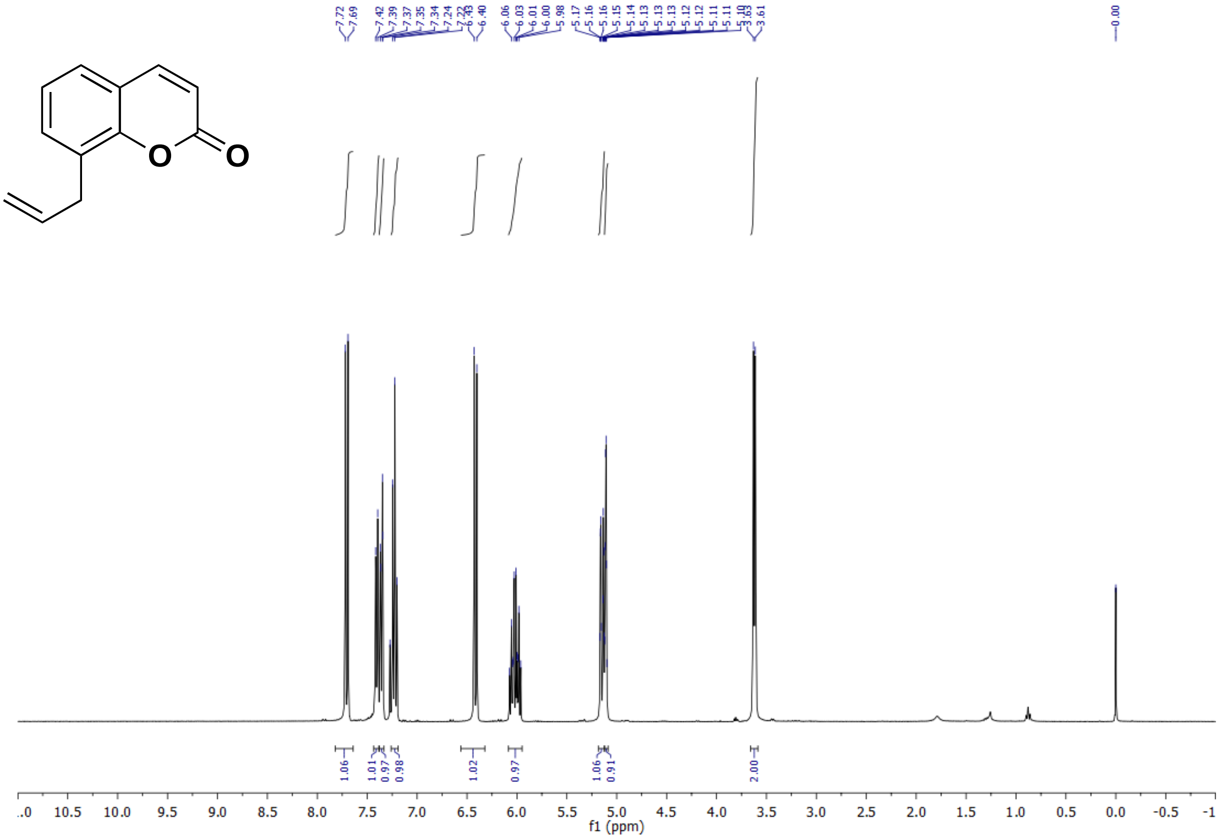

^{13}C NMR (90 MHz, $CDCl_3$) of 8-allyl-2*H*-chromen-2-one (4g)

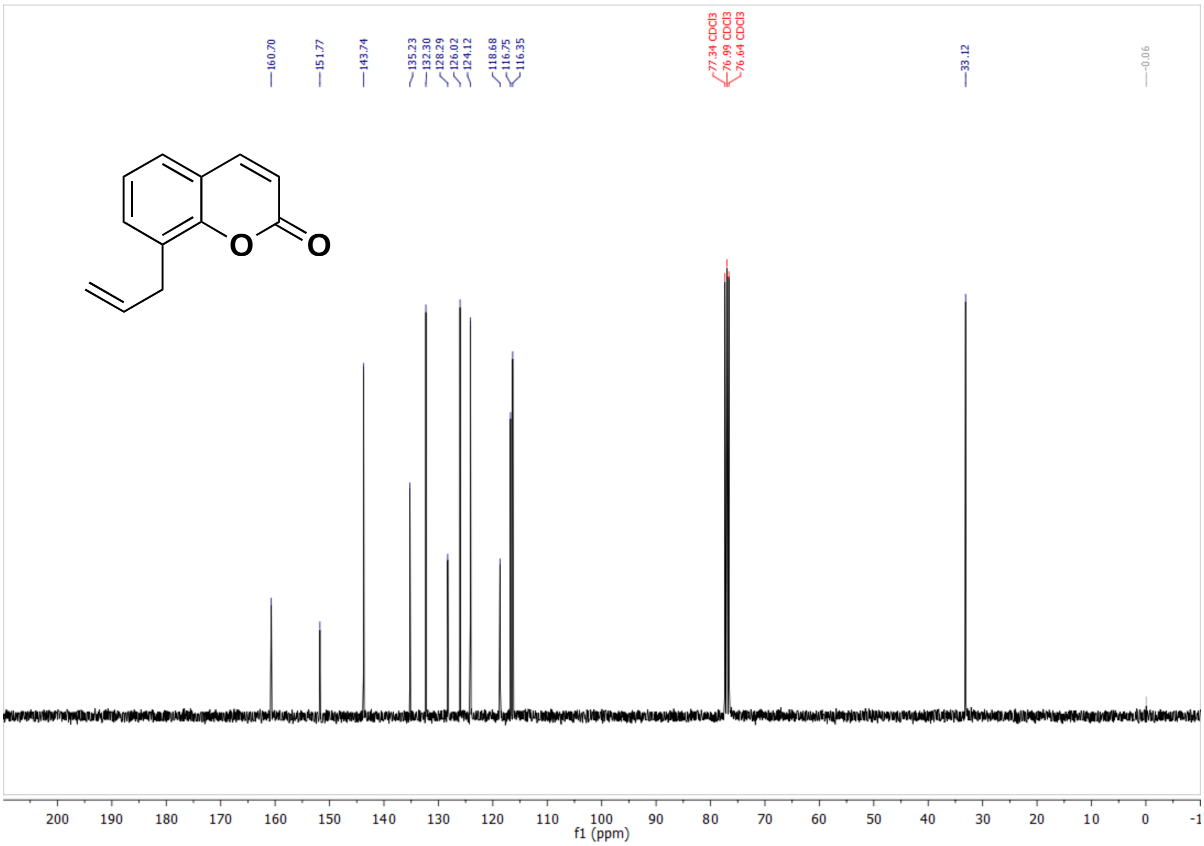

^{1}H NMR (360 MHz, $CDCl_3$) of 6-bromo-2*H*-chromen-2-one (4h)

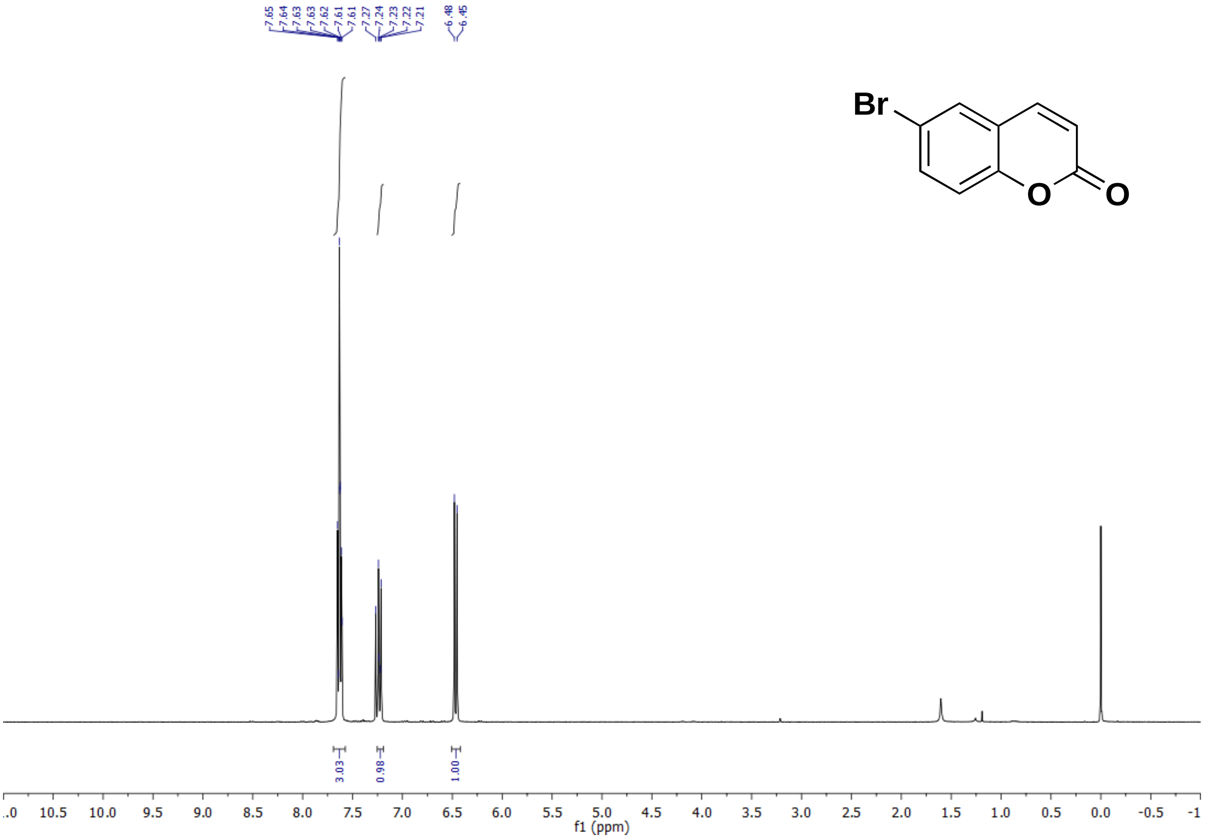

^{13}C NMR (90 MHz, $CDCl_3$) of 6-bromo-2*H*-chromen-2-one (4h)

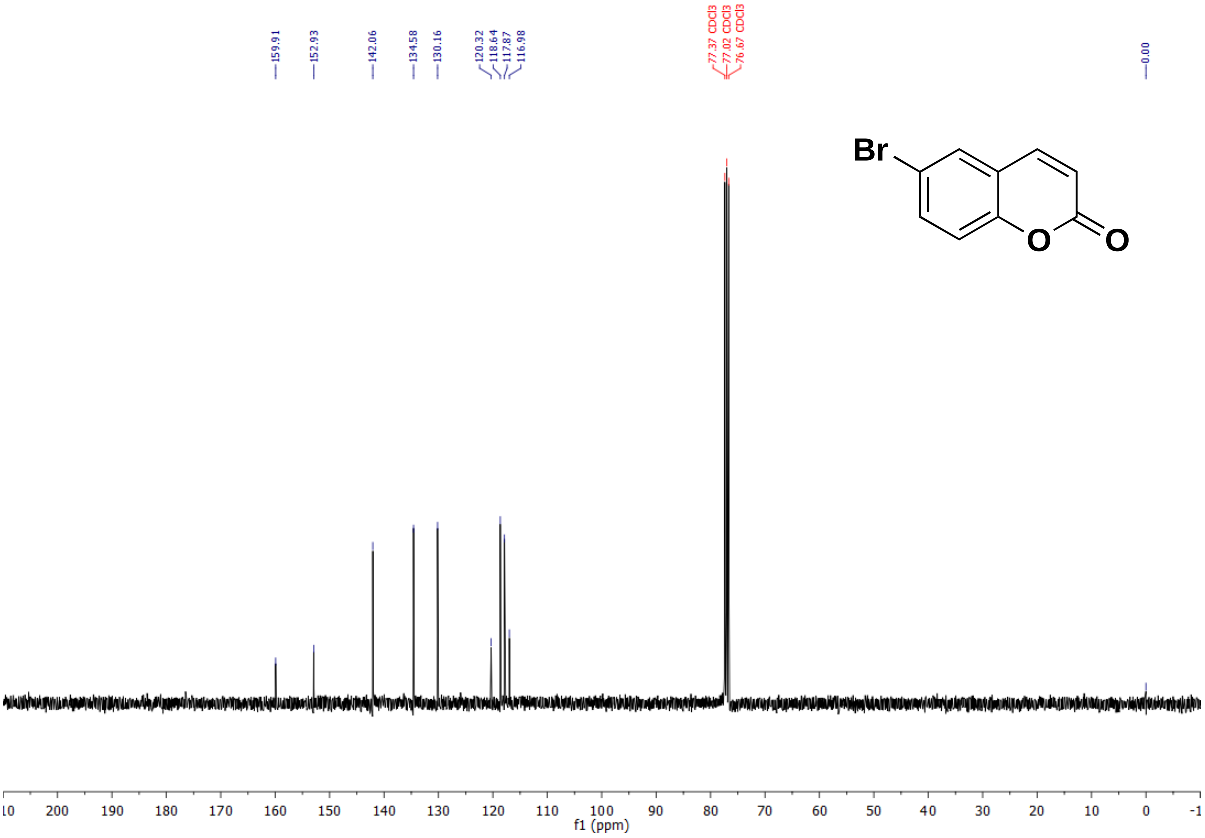

^{1}H NMR (360 MHz, $CDCl_3$) of 6,8-di-*tert*-butyl-2*H*-chromen-2-one (4i)

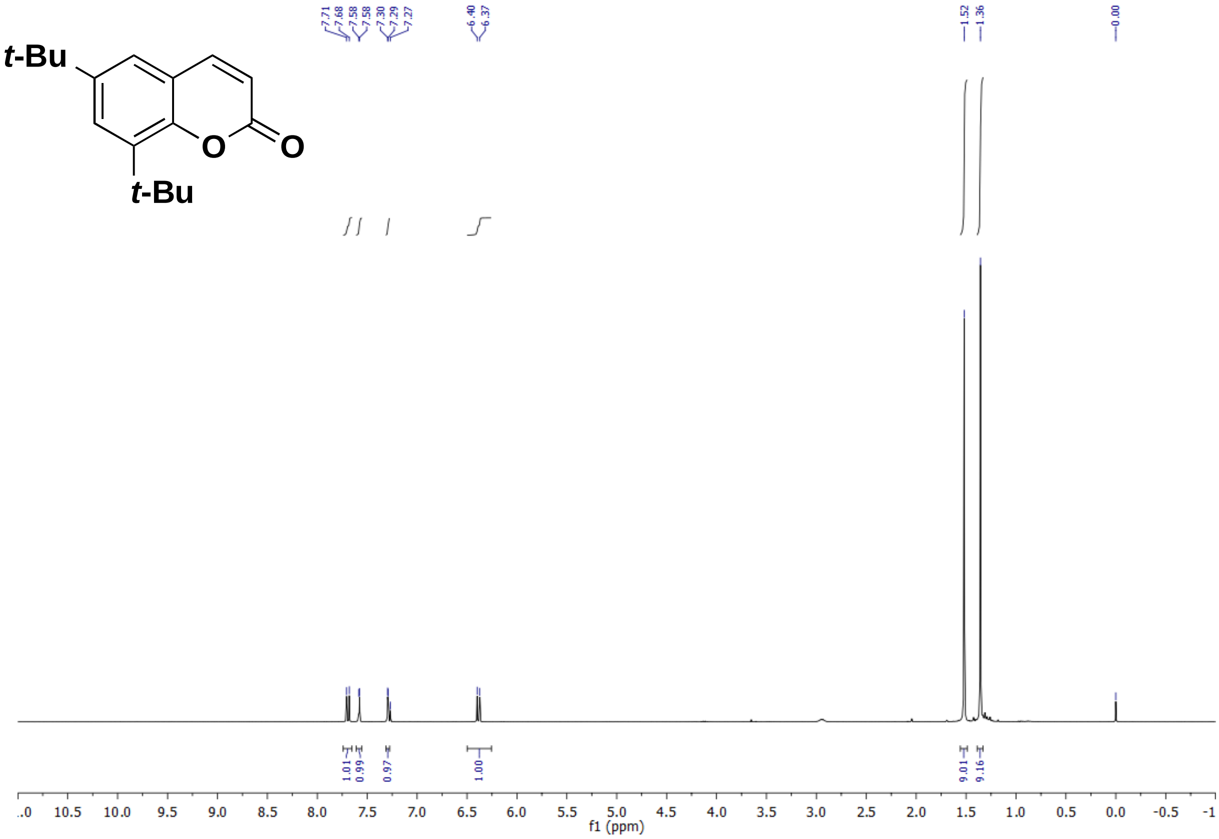

^{13}C NMR (90 MHz, $CDCl_3$) of 6,8-di-*tert*-butyl-2*H*-chromen-2-one (4i)

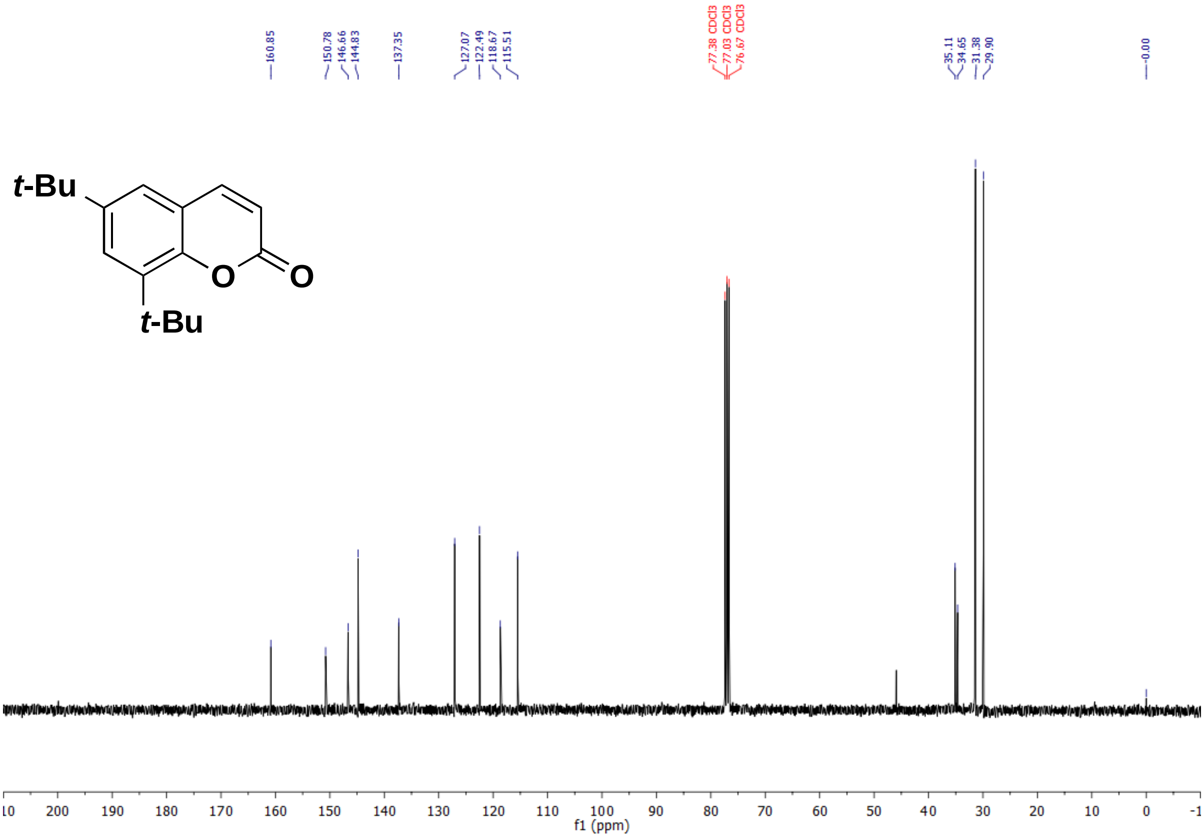

Supplement: File 1 — Experimental procedures, characterization data and copies of NMR spectra. [file Beilstein_J_Org_Chem-08-1630-s001.pdf]
